# Boundary regularity of an isotropically censored nonlocal operator

**DOI:** 10.1007/s00526-023-02544-0

**Published:** 2023-09-01

**Authors:** Hardy Chan

**Affiliations:** Department of Mathematics and Computer Science, University of Basel, Spiegelgasse 1, 4051 Basel, Switzerland

**Keywords:** 35B65, 35R11

## Abstract

In a bounded domain, we consider a variable range nonlocal operator, which is maximally isotropic in the sense that its radius of interaction equals the distance to the boundary. We establish $C^{1,\alpha }$ boundary regularity and existence results for the Dirichlet problem.

## Introduction

### General setting and the operator

Let $n\ge 1$ and $\Omega \subset \mathbb {R}^n$ be a bounded, connected domain of class $C^{1,1}$. Let $d_{\Omega }:{\overline{\Omega }}\rightarrow [0,+\infty )$ be the distance to the boundary,

$$\begin{aligned} d_{\Omega }(x)={{\,\textrm{dist}\,}}(x,\partial \Omega ). \end{aligned}$$

When no confusion arises, we simply write $d=d_\Omega$. It is convenient to smooth out the distance function by taking $\delta \in C^{1,1}(\overline{\Omega })$ such that $\delta =d$ when $d<d_0$, for a small $d_0>0$. A similar notation is employed for other domains.

Let $s\in (0,1)$. Write $B_r(x)=\left\{ y\in \mathbb {R}^n:|y-x|<r\right\}$ and $B_r=B_r(0)$. We introduce the operator

$$\begin{aligned}\begin{aligned} \mathcal {L}_\Omega u(x)&= C_{n,s} d(x)^{2s-2} \text {P.V.}\,\int _{B_{d(x)}(x)} \dfrac{u(x)-u(y)}{|x-y|^{n+2s}} \,dy\\&= \dfrac{C_{n,s}}{2} d(x)^{2s-2} \int _{B_{d(x)}} \dfrac{2u(x)-u(x+y)-u(x-y)}{|y|^{n+2s}} \,dy, \end{aligned}\end{aligned}$$

where the normalization constant is given by

1.1 $$\begin{aligned} C_{n,s}^{-1}= & {} \dfrac{1}{2}r^{2s-2} \int _{B_r} \dfrac{ y_n^2 }{ |y|^{n+2s} } \,dy, \quad \forall r>0, \quad \text { or }\nonumber \\ C_{n,s}= & {} \dfrac{2n(2-2s)}{|\mathbb {S}^{n-1}|} =4(1-s)\dfrac{ \Gamma (\frac{n+2}{2}) }{ \pi ^{\frac{n}{2}} }. \end{aligned}$$

This is an isotropic regional fractional Laplacian, and the factor $d(x)^{2s-2}$ is inserted to ensure that $\mathcal {L}_\Omega u(x)$ converges as $x\rightarrow \partial \Omega$ to a nontrivial limit, namely $-\Delta u(x)$. See Lemma A.1.

Probabilistically speaking, the operator $\mathcal {L}_\Omega$ generates a Lévy type process where a particle at $x\in \Omega$ jumps randomly and isotropically in the largest possible ball $B_{d(x)}(x)$ contained inside $\Omega$.

We list some characteristic properties and consequences.

- $\mathcal {L}_\Omega$ enjoys a mid-range maximum principle Proposition 2.2, whose strength lies between the local one for $-\Delta$ and the global one for $(-\Delta )^s$: no absolute minima exist in the region where $\mathcal {L}_\Omega u\ge 0$, provided that *u* is non-negative in the domain of interaction outside of that region. As a result, local barriers suffice to control boundary behaviors.
- The domain of interaction depends on the point of evaluation. Thus, extra effort is needed in the construction of barriers in Sect. 3, even following the established idea [11].
- $\mathcal {L}_{\Omega }$ is of order 2*s* but scales quadratically and satisfies the classical Hopf lemma, see Lemma A.4 and Lemma 4.6.
- $\mathcal {L}_\Omega$ is not variational. To see this, take $\Omega =(0,1)$. Upon integrating by parts, one immediate sees “hidden boundary terms” at $x=1/2$. In higher dimensions the “hidden boundary” can be thought of as points having at least two projections to the boundary. Unfortunately, all these points contribute in such a different way that the resulting expression would not be manageable. Consequently, no weak formulations due to integration by parts can be expected. Existence is to be established in the viscosity sense, in Sect. 8.

Generic and universal constants are denoted by *C*, *c*. They depend only on *n*, *s* and $\Omega$.

### Main results

Consider the Dirichlet problem

1.2 $$\begin{aligned} {\left\{ \begin{array}{ll} \mathcal {L}_\Omega u=f &{} \text { in }\Omega ,\\ u=0 &{} \text { on }\partial \Omega . \end{array}\right. }\end{aligned}$$

We study the regularity properties of its classical solutions *u*, meaning that $u\in C^{2s+}(\Omega )\cap C({\overline{\Omega }})$, where $C^{2s+}(\Omega )=\bigcup _{\beta >0}C^{2s+\beta }(\Omega )$.

Our main results are the following.

#### Theorem 1.1

(A priori regularity up to boundary) Suppose $\Omega$ is a bounded domain of class $C^{1,1}$ in $\mathbb {R}^n$ with $n\ge 1$, $s\in (0,1)$ and $u\in C^{2\,s+}(\Omega )\cap C({\overline{\Omega }})$ solves (1.2). Then there exists $\alpha _0=\alpha _0(n,s)\in (0,1)$ such that for any $\alpha \in (0,\alpha _0)$, the following holds. If either

1. $$s\in (\frac{1}{2},1)$$, $$f\in L^\infty (\Omega )$$; or
2. $$s\in (0,\frac{1}{2}]$$, $$f\in C^{\alpha +1-2s}(\Omega )$$,

then $$u\in C^{1,\alpha }(\overline{\Omega })$$, with

$$\begin{aligned} \left\| {u}\right\| _{C^{1,\alpha }(\overline{\Omega })}\le C. \end{aligned}$$

Here *C* depends on $n,s,\Omega$ and the corresponding norm of *f*.

Higher regularity up to the boundary remains open. There are two difficulties:

- The condition $\alpha <1$ is crucially used in the proof of Proposition 6.1, where the quadratic growth (linear in $x'$ times linear in $x_n$) contradicts the control $|x|^{1+\alpha }$.
- A complete study of the action of $\mathcal {L}_{\mathbb {R}^n_+}$ on monomials is missing (Lemma 4.3).

#### Theorem 1.2

(Existence) Suppose $\Omega$ is a bounded domain of class $C^{1,1}$ in $\mathbb {R}^n$ with $n\ge 1$, $s,\beta \in (0,1)$ so that $\beta +2s$ is not an integer, $f\in C^\beta (\Omega )$. Then there exists a unique $u\in C^{2s+\beta }(\Omega )\cap C(\overline{\Omega })$ solving (1.2). Moreover, if $\beta >(1-2s)_+$, then $u\in C^{1,\alpha }(\overline{\Omega })$, for some $\alpha \in (0,2\,s+\beta -1)$ determined by Theorem 1.1.

#### Remark 1.3

Crucial to construction of the barrier (Proposition 3.1) is the uniform exterior ball condition. This is satisfied by bounded domains of class $C^{1,1}$, as well as their blow-ups around a boundary point.

### Main ideas

Let us explain the heuristics of the proof. By definition, *u* is $C^{1,\alpha }$ at $0\in \partial \Omega$ if

$$\begin{aligned} u(x) =c_0(x\cdot \nu ) +O(|x|^{1+\alpha }), \quad \text { as }x\rightarrow 0. \end{aligned}$$

This is implied (see Lemma C.1) by the expansion

1.3 $$\begin{aligned} \dfrac{u(x)}{d(x)} =c_0+O(|x|^{\alpha }), \quad \text { as }x\rightarrow 0, \end{aligned}$$

i.e. *u*/*d* is $C^{\alpha }$ up to $0\in \partial \Omega$. By building suitable barriers in Sect. 3, we will be able to obtain global Hölder regularity (Proposition 5.4). This allows the use of a blow-up argument to reduce the problem to a half plane.

It then suffices to prove a Liouville theorem (Proposition 6.1) for solutions to the homogeneous equation in the half space, namely

$$\begin{aligned}{\left\{ \begin{array}{ll} \mathcal {L}_{\mathbb {R}^n_+}u=0 &{} \text { in }\mathbb {R}^n_+,\\ u=0 &{} \text { on }\partial \mathbb {R}^n_+, \end{array}\right. }\end{aligned}$$

under the growth $u(x)=O(|x|^{1+\alpha })$: the only solution is $u=cx_n$. Since the homogeneous equation is preserved under scaling and tangential differentiations, solutions can be shown to be one dimensional (1D) (e.g. Lemma 6.4). Hence, we need to show that solutions $u(x',x_n)$, which is independent of $x'$, to

$$\begin{aligned}{\left\{ \begin{array}{ll} \mathcal {L}_{\mathbb {R}_+^n}u(x_n)=0 &{} \text { for } x_n\in \mathbb {R}_+,\\ u(0)=0, \end{array}\right. }\end{aligned}$$

which grow no faster than $x_n^{1+\alpha }$ must be linear (Lemma 6.2). This is in turn implied by the boundary regularity in the half-line (Proposition 4.1), namely

$$\begin{aligned} \dfrac{u(x_n)}{x_n}\in C^{\alpha } \quad \text { up to } 0. \end{aligned}$$

To show this, one simply proves a boundary Harnack inequality (Lemma 4.7) and an improvement of oscillation (Lemma 4.8).

### Related works

The boundary Harnack inequality for a nonlocal elliptic operator in non-divergence form is proved by Ros-Oton–Serra in [12, Theorem 1.2]. A similar type of nonlocal operator with fixed horizon (range of interaction) at every point has been considered by Bellido and Ortega [6].

### Generalizations

We expect that the techniques introduced in this paper should be able to prove similar results in the parabolic setting, and when $\mathcal {L}_{\Omega }$ is replaced by an analogous integro-differential operator of order 2*s* with any homogeneous kernel.

## Preliminary results

Clearly $\mathcal {L}_\Omega$ satisfies the global maximum principle.

### Lemma 1.4

(Global maximum principle) Suppose $u\in C^{2\,s+}(\Omega )\cap C({\overline{\Omega }})$ solves

2.1 $$\begin{aligned} {\left\{ \begin{array}{ll} \mathcal {L}_\Omega u\ge 0 &{} \text { in }\Omega ,\\ u\ge 0 &{} \text { on }\partial \Omega . \end{array}\right. } \end{aligned}$$

Then either $u\equiv 0$ in ${\overline{\Omega }}$ or $u>0$ in $\Omega$.

### Proof

At any interior minimum $x_0$, $\mathcal {L}_\Omega u(x_0)\le 0$, with strict inequality unless $u\equiv u(x_0)$ in $B_{d(x_0)}(x_0)$. But the latter ball contains a sequence converging to $\partial \Omega$ where $u\ge 0$. $\square$

A more careful examination yields the following version of maximum principle. It is especially useful to study the blow-up equation when the domain becomes unbounded.

### Proposition 1.5

(Mid-range strong maximum principle) Let ${U}\subset \mathbb {R}^n$ be a domain that is not necessarily bounded. Suppose *G* is non-empty, bounded, open in *U*. The domain interacting with *G* is

2.2 $$\begin{aligned} G_*=\bigcup _{y\in G} B_{d_{U}(y)}(y) \ne \varnothing . \end{aligned}$$

Suppose $u\in C^{2s+}(G)\cap C(\overline{G_*})$, is a solution to

$$\begin{aligned}{\left\{ \begin{array}{ll} \mathcal {L}_U u \ge 0 &{} \text { in }G,\\ u\ge 0 &{} \text { in }\overline{G_*}\setminus G. \end{array}\right. }\end{aligned}$$

Then $$u\ge 0$$ in *G*.

### Proof

If $\min _{\overline{G}}u$ is attained on $\partial G \subset \overline{G_*}{\setminus } G$ and not in *G*, then $u\ge 0$ in *G* by the Dirichlet condition. If $u(x_0)=\min _{G}u\le 0$, then

$$\begin{aligned}\begin{aligned} 0&\le \mathcal {L}_U u(x_0)\\&= C_{n,s} d_U(x_0)^{2s-2}\, \text {P.V.}\,\int _{B_{d_{U}(x_0)}(x_0)} \dfrac{ u(x_0)-u(y) }{ |x_0-y|^{n+2s} } \,dy\\&\le C_{n,s} d_U(x_0)^{2s-2}\,\\&\left( \text {P.V.}\,\int _{B_{d_U(x_0)}(x_0) \cap G} \dfrac{ u(x_0)-u(y) }{ |x_0-y|^{n+2s} } \,dy +u(x_0) \int _{ B_{d_{U}(x_0)}(x_0) \setminus G } \dfrac{ 1 }{ |x_0-y|^{n+2s} } \,dy \right) \\&\le 0. \end{aligned}\end{aligned}$$

Thus equality holds and $u\equiv u(x_0)=0$ in *G*. $\square$

The interior Harnack inequality is known to DiCastro–Kuusi–Palatucci [2].

### Lemma 1.6

(Interior Harnack inequality) Suppose $B_r(z)\subset \Omega \subset \mathbb {R}^n$, $n\ge 1$. If $u\ge 0$ in $B_r(z)_*$ (as defined in (2.2)) and

$$\begin{aligned} \mathcal {L}_{\Omega } u=0 \quad \text { in }B_r(z), \end{aligned}$$

then for any $\eta >0$, there exists $C_0(n,s,\Omega ,\eta )>0$ such that

$$\begin{aligned} u(x)\le C_0(\eta ) u(y) \quad \forall x,y\in B_{(1-\eta )r}(z). \end{aligned}$$

Now we state the interior estimates which follows from the corresponding result for the restricted fractional Laplacian [9, 10]. We denote

2.3 $$\begin{aligned} \left\| {u}\right\| _{L^1_{\mu }(\Omega )} =\int _{\Omega } \dfrac{ |u(y)| }{ 1+|y|^{n+\mu } } \,dy, \quad \text { for }\mu \in \mathbb {R}. \end{aligned}$$

### Lemma 1.7

(Interior estimates) Suppose $U\subset \mathbb {R}^n$ is not necessarily bounded, and $B_1\subset B_4\subset U$. Suppose $u\in C^{2\,s+}(\overline{B_1})\cap C({\overline{U}})$ is a solution to

$$\begin{aligned} \mathcal {L}_{U} u=f \quad \text { in }B_1, \end{aligned}$$

for $f\in L^\infty (B_1)$. Then there exists a constant $C=C(n,s,\epsilon )>0$ such that

$$\begin{aligned}{\left\{ \begin{array}{ll} \left\| {u}\right\| _{C^{2s}(\overline{B_{1/2}})} \\ \le C\left( \left\| {u}\right\| _{L^\infty (B_1)} +\left\| {u}\right\| _{L^1_{2s}(U)} +\left\| {d_U}\right\| _{L^\infty (B_1)}^{2-2s} \left\| {f}\right\| _{L^\infty (B_1)} \right) &{} \text { for }s\ne \frac{1}{2},\\ \left\| {u}\right\| _{C^{1-\epsilon }(\overline{B_{1/2}})}\\ \le C\left( \left\| {u}\right\| _{L^\infty (B_1)} +\left\| {u}\right\| _{L^1_{2s}(U)} +\left\| {d_U}\right\| _{L^\infty (B_1)}^{2-2s} \left\| {f}\right\| _{L^\infty (B_1)} \right) &{} \text { for }s=\frac{1}{2}. \end{array}\right. }\end{aligned}$$

Moreover, if $f\in C^\beta (\overline{B_1})$ for $\beta \in (0,1)$ and $\beta +2s$ is not an integer, then there exists $C=C(n,s,\beta )>0$ such that

$$\begin{aligned}{} & {} \left\| {u}\right\| _{C^{2s+\beta }(\overline{B_{1/2}})} \\{} & {} \le C\left( \left\| {u}\right\| _{C^\beta (\overline{B_1})} +\left\| {u}\right\| _{L^\infty (B_{d_1})} +\left\| {u}\right\| _{L^1_{2s}(U)} +\left\| {d_U}\right\| _{L^\infty (B_1)}^{2-2s} \left\| {f}\right\| _{C^\beta (\overline{B_1})} \right) , \end{aligned}$$

where $$d_1=\left\| {d_U}\right\| _{L^\infty (B_1)}+2$$.

### Remark 2.5

We notice that, because the operator $\mathcal {L}_U$ degenerates away from the boundary, so does the estimate (through the term $\left\| {d_U}\right\| _{L^\infty (B_1)}^{2-2s}$).

### Proof

Let us extend *u* by zero outside *U*. In this proof we write $d=d_U$. At each interior point $x\in U$, one can rewrite the equation in terms of the restricted fractional Laplacian, namely

$$\begin{aligned} (-\Delta )^su=g[u](x) \quad \text { in }B_1, \end{aligned}$$

where

$$\begin{aligned} g[u](x) = \dfrac{ c_{n,s} }{ C_{n,s} } \left( C_{n,s}\int _{B_{d(x)}^c} \dfrac{ u(x)-u(x+y) }{ |y|^{n+2s} } \,dy +f(x)d(x)^{2-2s} \right) , \end{aligned}$$

with $c_{n,s}=2^{2s}\pi ^{-\frac{n}{2}}\Gamma (\frac{n+2s}{2})/|\Gamma (-s)|$ being the normalization constant for $(-\Delta )^s$. We first prove the $C^{2s}$ (or $C^{1-\epsilon }$) regularity. Note that for $x\in B_1$, we have $d(x)\ge 3$ and $B_{d(x)}(x)^c\subset B_1^c$, so

$$\begin{aligned}\begin{aligned} |g[u](x)|&\le C\left( d(x)^{-2s}|u(x)| +\int _{B_1^c} \dfrac{|u(y)|}{|y|^{n+2s}} \,dy +d(x)^{2-2s}|f(x)| \right) \\&\le C\left( \left\| {u}\right\| _{L^\infty (B_1)} +\left\| {u}\right\| _{L^1_{2s}(U)} +\left\| {d}\right\| _{L^\infty (B_1)}^{2-2s} \left\| {f}\right\| _{L^\infty (B_1)} \right) . \end{aligned}\end{aligned}$$

Suppose first $s\ne \frac{1}{2}$. By [10, Theorem 1.1(a)],

$$\begin{aligned}\begin{aligned} \left\| {u}\right\| _{C^{2s}(\overline{B_{1/2}})}&\le C\left( \left\| {u}\right\| _{L^\infty (B_1)} +\left\| {u}\right\| _{L^1_{2s}(U)} +\left\| {g}\right\| _{L^\infty (B_1)} \right) \\&\le C\left( \left\| {u}\right\| _{L^\infty (B_1)} +\left\| {u}\right\| _{L^1_{2s}(U)} +\left\| {d}\right\| _{L^\infty (B_1)}^{2-2s} \left\| {f}\right\| _{L^\infty (B_1)} \right) . \end{aligned}\end{aligned}$$

When $s=\frac{1}{2}$, using again [10, Theorem 1.1(a)], we replace accordingly the $C^{2s}$ norm by $C^{1-\epsilon }$ norm for any $\epsilon \in (0,1)$, with the constant depending also on $\epsilon$.

Now we prove the higher regularity. Up to multiplicative constants, we decompose $g=g_1+g_2+g_3$ where

$$\begin{aligned}\begin{aligned} g_1(x)&=d(x)^{2-2s}f(x)\\ g_2(x)&=d(x)^{-2s}u(x)\\ g_3(x)&=\int _{U\cap B_{d(x)}(x)^c} \dfrac{u(z)}{|z-x|^{n+2s}}\,dz. \end{aligned}\end{aligned}$$

Since $\left[ {\varphi ^p}\right] _{C^\beta }\le p \left\| {\varphi ^{p-1}}\right\| _{L^\infty }\left[ {\varphi }\right] _{C^\beta }$ for $\beta \in (0,1)$ and $p\in \mathbb {R}$, we use the bounds $3\le d(x)\le {{\,\textrm{inrad}\,}}(U)$ and $\left\| {d}\right\| _{C^{0,1}({\overline{U}})}\le 1$ to control

$$\begin{aligned}\begin{aligned} \left[ {g_1}\right] _{C^\beta (\overline{B_1})}&\le C\left( \left\| {d}\right\| _{L^\infty (B_1)}^{2-2s} \left[ {f}\right] _{C^\beta (\overline{B_1})} +\left\| {d}\right\| _{L^\infty (B_1)}^{1-2s} \left\| {f}\right\| _{L^\infty (B_1)} \right) \\&\le C\left\| {d}\right\| _{L^\infty (B_1)}^{2-2s} \left\| {f}\right\| _{C^\beta (\overline{B_1})},\\ \left[ {g_2}\right] _{C^\beta (\overline{B_1})}&\le C\left\| {u}\right\| _{C^\beta (\overline{B_1})}. \end{aligned}\end{aligned}$$

For $x,y\in \overline{B_1}$, we express

$$\begin{aligned}\begin{aligned} g_3(x)-g_3(y)&=\int _{U\cap B_{d(x)}(x)^c} u(z)\left( \dfrac{1}{|z-x|^{n+2s}} -\dfrac{1}{|z-y|^{n+2s}} \right) \,dz\\&\quad \; +\left( \int _{B_{d(x)}(x)^c} -\int _{B_{d(y)}(y)^c} \right) \dfrac{u(z)}{|z-y|^{n+2s}} \,dz. \end{aligned}\end{aligned}$$

For the integral in the first line, note that $3\le d(x)\le |z|$. By mean value theorem, there exists $x_*\in B_1$ such that

$$\begin{aligned}\begin{aligned} \left| \int _{U\cap B_{d(x)}(x)^c} u(z)\left( \dfrac{1}{|z-x|^{n+2s}} -\dfrac{1}{|z-y|^{n+2s}} \right) \,dz \right|&\le C \int _{U\cap B_{d(x)}(x)^c} \dfrac{ |u(z)||x-y| }{ |z-x_*|^{n+2s+1} } \,dz\\&\le C\left\| {u}\right\| _{L^1_{2s+1}(U)} |x-y|. \end{aligned}\end{aligned}$$

For the second line we note that there is a nontrivial contribution only in the symmetric difference $B_{d(x)}(x)^c \triangle B_{d(y)}(y)^c$, which lies in an annulus of width at most of order $|x-y|$. More precisely, we have

$$\begin{aligned} B_1 \subset B_{\frac{d(x)+d(y)}{2}-|x-y|} \subset B_{d(x)}(x)^c \triangle B_{d(y)}(y)^c \subset B_{\frac{d(x)+d(y)}{2}+|x-y|}. \end{aligned}$$

Therefore, using $|z|\ge d(x) \ge 3 \ge 3|y|$ and ${{\,\textrm{supp}\,}}u\subset {\overline{\Omega }}$,

$$\begin{aligned}\begin{aligned} \left| \left( \int _{B_{d(x)}(x)^c} -\int _{B_{d(y)}(y)^c} \right) \dfrac{u(z)}{|z-y|^{n+2s}} \,dz \right|&\le \int _{ \frac{d(x)+d(y)}{2}-|x-y| \le |z| \le \frac{d(x)+d(y)}{2}+|x-y| } \dfrac{ |u(z)| }{ |z|^{n+2s} }\,dz\\&\le C\dfrac{ \left\| {u}\right\| _{L^\infty (B_{(d(x)+d(y))/2+|x-y|})} }{ (\frac{d(x)+d(y)}{2})^{1+2s} } |x-y|\\&\le C\left\| {u}\right\| _{L^\infty (B_{d(x)\vee d(y)+2})} |x-y|\\&\le C\left\| {u}\right\| _{L^\infty (B_{d_1})} |x-y|, \end{aligned}\end{aligned}$$

where $d_1:=\left\| {d}\right\| _{L^\infty (B_1)}+2$. In summary,

$$\begin{aligned} \left[ {g}\right] _{C^\beta (\overline{B_1})}\le & {} C\bigg ( \left\| {u}\right\| _{C^\beta (\overline{B_1})} +\left\| {u}\right\| _{L^\infty (B_{d_1})} +\left\| {u}\right\| _{L^1_{2s+1}(U)}\\{} & {} +\left\| {d}\right\| _{L^\infty (B_1)}^{2-2s} \left[ {f}\right] _{C^\beta (\overline{B_1})} +\left\| {d}\right\| _{L^\infty (B_1)}^{1-2s} \left\| {f}\right\| _{L^\infty (B_1)} \bigg ).\\ \left\| {g}\right\| _{C^\beta (\overline{B_1})}\le & {} C\bigg ( \left\| {u}\right\| _{C^\beta (\overline{B_1})}+\left\| {u}\right\| _{L^\infty (B_{d_1})}\\{} & {} +\left\| {u}\right\| _{L^1_{2s+1}(U)} +\left\| {d}\right\| _{L^\infty (B_1)}^{2-2s} \left\| {f}\right\| _{C^\beta (\overline{B_1})} \bigg ). \end{aligned}$$

Now, by [9, Corollary 2.4],

$$\begin{aligned} \begin{aligned} \left\| {u}\right\| _{C^{\beta +2s}(\overline{B_{1/2}})}&\le C\left( \left\| {u}\right\| _{C^\beta (\overline{B_1})} +\left\| {u}\right\| _{L^1_{2s}(U)} +\left\| {g}\right\| _{C^\beta (\overline{B_1})} \right) \\&\le C\left( \left\| {u}\right\| _{C^\beta (\overline{B_1})}+\left\| {u}\right\| _{L^\infty (B_{d_1})} +\left\| {u}\right\| _{L^1_{2s}(U)}\right. \\&\left. \qquad +\left\| {d}\right\| _{L^\infty (B_1)}^{2-2s} \left\| {f}\right\| _{C^\beta (\overline{B_1})} \right) , \end{aligned} \end{aligned}$$

where we have absorbed $\left\| {u}\right\| _{L^1_{2s+1}(U)}$ by $\left\| {u}\right\| _{L^1_{2s}(U)}$. $\square$

## The barriers

### Super-solution near the boundary

We construct a barrier in the spirit of [10]. Since the domain of interaction varies from point to point, we must compute at all points.

The idea is to consider powers of the distance function to a ball which touches the domain from the outside, since we want the super-solution to be strictly positive except at the contact point, however near the boundary.

#### Proposition 1.9

(Super-solution) Let ${U}\subset \mathbb {R}^n$ be a possibly unbounded domain of class $C^{1,1}$. Suppose $x_0\in \partial {U}$ can be touched by an exterior ball of radius $b>0$. Then, there exists a constant $r_0>0$ and a function $\varphi ^{(x_0)}\in C^2 \bigl ({U\cap B_{2r_0}(x_0)}\bigr ) \cap C^{0,1}\bigl (\overline{U\cap B_{2r_0}(x_0)}\bigr )$ satisfying

$$\begin{aligned}{\left\{ \begin{array}{ll} \mathcal {L}_{{U}}\varphi ^{(x_0)} \ge 1 &{} \text { in }{U} \cap B_{r_0}(x_0),\\ \varphi ^{(x_0)} \ge 1 &{} \text { in }{U} \cap \bigl (B_{2r_0}(x_0)\setminus B_{r_0}(x_0)\bigr ),\\ \varphi ^{(x_0)} \ge 0 &{} \text { in }\overline{U \cap B_{2r_0}(x_0)},\\ \varphi ^{(x_0)} \le Cd_U &{} \text { on }U\cap B_{r_0}(x_0) \cap (\mathbb {R}\nu (x_0)),\\ \varphi ^{(x_0)}(x_0)=0. \end{array}\right. }\end{aligned}$$

Here $\nu (x_0)$ is the (outer) normal at $x_0\in \partial \Omega$, and $r_0$ and *C* depend only on *n*, *s* and *b*.

Upon a translation and a rotation, the exterior ball condition states that $B_b(-be_n)$ touches $\partial {U}$ at $x_0=0\in \partial {U}$ from the outside.

The super-solution will be built from

$$\begin{aligned} d_{B_b(-be_n)}(y)^p =(d_{-be_n}(y)-b)^p =(|y+be_n|-b)^p, \end{aligned}$$

for $p=1$ and $p\sim 2^-$. As in [10], at each point $x\in {U}$ we compare $d_{B_b(-be_n)}(y)^p$ with the one-dimensional function

$$\begin{aligned} d_{\mathcal {T}_x}(y)^p =(d_{\mathcal {P}_x}(y)-b)^p =\bigl ( (y+be_n)\cdot {\varvec{v}} -b \bigr )^p, \end{aligned}$$

where

$$\begin{aligned} \mathcal {T}_x:= & {} \left\{ y\in \mathbb {R}^n:(y+be_n)\cdot {\varvec{v}}=b\right\} \\ \mathcal {P}_x:= & {} \left\{ y\in \mathbb {R}^n:(y+be_n)\cdot {\varvec{v}}=0\right\} \end{aligned}$$

are the hyperplanes which are orthogonal to

$$\begin{aligned} {\varvec{v}}={\varvec{v}}_x:=\dfrac{x+be_n}{|x+be_n|} \end{aligned}$$

and contain respectively $-be_n+b{\varvec{v}}$ and $-be_n$.

First we consider the planar barrier. This uses the fact that $-\mathcal {L}_{U}$ behaves like the Laplacian near $\partial {U}$. (Notice that this computation is valid for $d_{\mathcal {T}_x}^p$ only at the point *x*, although it is all we will need.)

#### Lemma 1.10

For $p>0$ and $x\in U$, we have

$$\begin{aligned} {-\mathcal {L}}_U\bigl ( d_{\mathcal {T}_x}^p \bigr )(x) = \bigl ( 1+O(|p-2|) \bigr ) d_{\mathcal {T}_x}(x)^{p-2}. \end{aligned}$$

Here the constant in $O(|p-2|)$ depends only on *n* and *s*.

#### Proof

Using $d_{\mathcal {T}_x}(x+ty)=d_{\mathcal {T}_x}(x)+ty\cdot {\varvec{v}}$ for $t\in [-1,1]$, we have

$$\begin{aligned}\begin{aligned}&\; {-\mathcal {L}}_{U}\bigl ( d_{\mathcal {T}_x}^p \bigr )(x)\\&\quad = \dfrac{C_{n,s}}{2} d_U(x)^{2s-2} \int _{|y|<d_U(x)} \dfrac{ \bigl ( d_{\mathcal {T}_x}(x) +y\cdot {\varvec{v}} \bigr )^p +\bigl ( d_{\mathcal {T}_x}(x) -y\cdot {\varvec{v}} \bigr )^p -2 d_{\mathcal {T}_x}(x) ^p }{ |y|^{n+2s} } \,dy\\&\quad = \dfrac{C_{n,s}}{2} d_U(x)^{2s-2} d_{\mathcal {T}_x}(x) ^p \int _{|y|<d_U(x)} \dfrac{ \bigl ( 1+\frac{y}{d_{\mathcal {T}_x}(x)} \cdot {\varvec{v}} \bigr )^p +\bigl ( 1-\frac{y}{d_{\mathcal {T}_x}(x)} \cdot {\varvec{v}} \bigr )^p -2 }{ |y|^{n+2s} } \,dy. \end{aligned}\end{aligned}$$

Changing variable to $y=d_{\mathcal {T}_x}(x)z$ (recall that $d_{\mathcal {T}_x}(x)>0$ for $x\ne 0\in \partial U$) and choosing another coordinate system for *z* such that ${\varvec{v}}$ is the direction of the last coordinate axis, we have (here $\psi$ is defined in (B.1))

3.1 $$\begin{aligned} \begin{aligned} {-\mathcal {L}}_{U}\bigl ( d_{\mathcal {T}_x}^p \bigr )(x)&= \dfrac{C_{n,s}}{2} d_{\mathcal {T}_x}(x) ^{p-2} \left( \dfrac{ d_U(x) }{ d_{\mathcal {T}_x}(x) } \right) ^{2s-2} \int _{ |z|<\frac{d_U(x)}{d_{\mathcal {T}_x}(x)} } \dfrac{ (1+z_n)^p +(1-z_n)^p -2 }{ |z|^{n+2s} } \,dz\\&= \psi \left( p,\dfrac{d_U(x)}{d_{\mathcal {T}_x}(x)} \right) d_{\mathcal {T}_x}(x)^{p-2}. \end{aligned}\end{aligned}$$

By Lemma B.1,

$$\begin{aligned} {-\mathcal {L}}_U\bigl ( d_{\mathcal {T}_x}^p \bigr )(x) = \psi \left( p,\dfrac{d_U(x)}{d_{\mathcal {T}_x}(x)} \right) d_{\mathcal {T}_x}(x)^{p-2} = \left( 1+O(|p-2|) \right) d_{\mathcal {T}_x}(x)^{p-2}, \end{aligned}$$

as desired. $$\square $$

Next we compare $d_{B_b(-be_n)}^p$ and $d_{\mathcal {T}_x}^p$ pointwise. For each fixed $x\in U$, hence ${\varvec{v}}\in \mathbb {S}^{n-1}$, we denote the projection of a vector $y\in \mathbb {R}^n$ onto $\mathcal {P}_x$ by

$$\begin{aligned} y'=y-(y\cdot {\varvec{v}}){\varvec{v}}. \end{aligned}$$

#### Lemma 1.11

For $$x\in U$$ and $$z\in B_{d_U(x)}(x)$$,

$$\begin{aligned} 0\le \bigl ( d_{B_b(-be_n)} -d_{\mathcal {T}_x} \bigr )(z) \le \dfrac{ |(z-x)'|^{2} }{ 2b }. \end{aligned}$$

#### Proof

For $z\in U$, we express

$$\begin{aligned}\begin{aligned} \bigl ( d_{B_b(-be_n)} -d_{\mathcal {T}_x} \bigr )(z)&= \bigl ( d_{B_{-be_n}}(z)-b \bigr ) -\bigl ( -d_{\mathcal {P}_x}(z)-b \bigr )\\&= \bigl ( |z+be_n|-b \bigr ) -\bigl ( (z+be_n)\cdot {\varvec{v}}-b \bigr )\\&= |z+be_n|-(z+be_n)\cdot {\varvec{v}}\\&= \sqrt{ \bigl ((z+be_n)\cdot {\varvec{v}}\bigr )^2 +|(z+be_n)'|^2 } -(z+be_n)\cdot {\varvec{v}}\\&= \frac{ |(z+be_n)'|^2 }{ \sqrt{ \bigl ((z+be_n)\cdot {\varvec{v}}\bigr )^2 +|(z+be_n)'|^2 } +(z+be_n)\cdot {\varvec{v}} }\\ \end{aligned}\end{aligned}$$

to see it is non-negative. We make the following observations:

- Since $$(x+be_n)'=0$$, $$(z+be_n)'=(z-x)'$$.
- The sum of radii of the interior disjoint balls $B_{d_U(x)}(x)$ and $B_b(-be_n)$ is at most the distance between the centers, giving $d_U(x)+b \le |x+be_n|$. This implies
  $$\begin{aligned} (z+be_n)\cdot {\varvec{v}} =(z-x)\cdot {\varvec{v}}+|x+be_n| \ge |x+be_n| - |z-x| \ge |x+be_n| - d_U(x) \ge b. \end{aligned}$$

Thus

$$\begin{aligned} \bigl ( d_{B_b(-be_n)}-d_{\mathcal {T}_x} \bigr )(z)&\le \frac{ |(z+be_n)'|^2 }{ 2(z+be_n)\cdot {\varvec{v}} } \le \frac{ |(z-x)'|^2 }{ 2b }. \end{aligned}$$

Now we can compute $\mathcal {L}_{U}(d_{-be_n}^p)$ locally near the boundary. $\square$

#### Lemma 1.12

Let $p\in [1,2]$ and $x\in U$. Then we have

3.2 $$\begin{aligned} {-\mathcal {L}}_U\bigl ( d_{B_b(-be_n)}^p \bigr )(x) \ge \bigl ( 1-C(2-p) \bigr ) d_{B_b(-be_n)}(x)^{p-2} \end{aligned}$$

and

3.3 $$\begin{aligned} \mathcal {L}_U(d_{B_b(-be_n)})(x) \ge -Cb^{-1}. \end{aligned}$$

Here *C* depends only on *n* and *s*.

#### Proof

We split

$$\begin{aligned}\begin{aligned} -\mathcal {L}_{U}\bigl ( d_{B_b(-be_n)}^p \bigr )(x) =-\mathcal {L}_{U}\bigl ( d_{B_b(-be_n)}^p-d_{\mathcal {T}_x}^p \bigr )(x) -\mathcal {L}_{U}\bigl ( d_{\mathcal {T}_x}^p \bigr )(x). \end{aligned}\end{aligned}$$

Since $\mathcal {T}_x$ is chosen such that $d_{B_b(-be_n)}(x)=d_{\mathcal {T}_x}(x)$ and $d_{B_b(-be_n)}\ge d_{\mathcal {T}_x}$ on $B_{d_U(x)}(x)$,

$$\begin{aligned}\begin{aligned}{} & {} -\mathcal {L}_{U}\bigl ( d_{B_b(-be_n)}^p-d_{\mathcal {T}_x}^p \bigr )(x) \\{} & {} = C_{n,s} d_U(x)^{2s-2}\, \text {P.V.}\,\int _{|y|<d_U(x)} \dfrac{ \bigl ( d_{B_b(-be_n)}^p-d_{\mathcal {T}_x}^{p} \bigr )(x+y) }{ |y|^{n+2s} } \,dy \ge 0. \end{aligned}\end{aligned}$$

Then (3.2) follows from Lemma 3.2.

For (3.3), since $d_{\mathcal {T}_x}$ is linear hence $\mathcal {L}_U$-harmonic, by Lemma 3.3 we have

$$\begin{aligned}\begin{aligned} -\mathcal {L}_{U}\bigl ( d_{B_b(-be_n)} \bigr )(x)&= -\mathcal {L}_{U}\bigl ( d_{B_b(-be_n)}-d_{\mathcal {T}_x} \bigr )(x)\\&= C_{n,s} d_U(x)^{2s-2}\, \text {P.V.}\,\int _{|y|<d_U(x)} \dfrac{ \bigl ( d_{B_b(-be_n)}-d_{\mathcal {T}_x} \bigr )(x+y) }{ |y|^{n+2s} } \,dy\\&\le Cb^{-1} d_U(x)^{2s-2}\, \text {P.V.}\,\int _{|y|<d_U(x)} \dfrac{ |y'|^{2} }{ |y|^{n+2s} } \,dy\\&\le Cb^{-1}. \end{aligned}\end{aligned}$$

We are ready to prove Proposition 3.1. $\square$

#### Proof of Proposition 3.1

Let ${\widetilde{\varphi }}(x)=2d_{B_b(-be_n)}(x)-d_{B_b(-be_n)}^{p}(x)$, where $p<2$ is chosen (using Lemma 3.4) such that

$$\begin{aligned} {-\mathcal {L}}_U\bigl ( d_{B_b(-be_n)}^p \bigr )(x) \ge \dfrac{1}{2} d_{B_b(-be_n)}(x)^{p-2}, \quad \text { for }x\in U. \end{aligned}$$

Then

$$\begin{aligned}\begin{aligned} {\mathcal {L}}_U{\widetilde{\varphi }}(x)&\ge -Cb^{-1} +\frac{1}{2} d_{B_b(-be_n)}(x)^{p-2} \ge 1, \end{aligned}\end{aligned}$$

whenever *x* is close enough to $B_b(-be_n)$, say $d_{B_b(-be_n)}(x)\le 2r_0<1$. On the other hand, we verify that

$$\begin{aligned}\begin{aligned} {\widetilde{\varphi }}(x) =d_{B_b(-be_n)}(x) \bigl ( 2-d_{B_b(-be_n)}(x)^{p-1} \bigr ) \ge d_{B_b(-be_n)}(x), \end{aligned}\end{aligned}$$

provided that $d_{B_b(-be_n)}(x)\le 1$. Moreover, on $\left\{ r_0 \le d_{B_b(-be_n)} \le 2r_0\right\} \cap U$ where $2r_0<1$,

$$\begin{aligned} {\widetilde{\varphi }}(x)\ge r_0. \end{aligned}$$

Therefore, $\varphi ^{(0)}=r_0^{-1}{\widetilde{\varphi }}$ is the desired super-solution. $\square$

### Super-solution for a bounded domain

A concave paraboloid serves as a simple global super-solution. Choose a coordinate system such that $0\in \Omega$. Let $M={{\,\textrm{diam}\,}}\Omega$ so that $\Omega \subset B_M$. Consider the positive, strictly concave function

$$\begin{aligned} \varphi ^{(1)}(x)=\dfrac{M^2-|x|^2}{2n}. \end{aligned}$$

When $\Omega =B_M$, this is known as the torsion function.

#### Lemma 1.13

There holds

$$\begin{aligned} {\left\{ \begin{array}{ll} \mathcal {L}_\Omega \varphi ^{(1)}=1 &{} \text { in }\Omega ,\\ \varphi ^{(1)}\ge 0 &{} \text { in }{\overline{\Omega }}. \end{array}\right. } \end{aligned}$$

#### Proof

For any $x\in \Omega$ and $y\in B_{d(x)}$, the parallelogram law implies

$$\begin{aligned} 2\varphi ^{(1)}(x) -\varphi ^{(1)}(x+y) -\varphi ^{(1)}(x-y) =\dfrac{|y|^2}{n}. \end{aligned}$$

Thus

$$\begin{aligned} \begin{aligned} \mathcal {L}_\Omega \varphi ^{(1)}(x)&=\dfrac{C_{n,s}}{2} d(x)^{2-2s} \int _{B_{d(x)}(0)} \dfrac{ 2\varphi ^{(1)}(x) -\varphi ^{(1)}(x+y) -\varphi ^{(1)}(x-y) }{ |y|^{n+2s} } \,dy\\&= \dfrac{C_{n,s}}{2n} d(x)^{2-2s} \int _{B_{d(x)}(0)} \dfrac{ |y|^2 }{ |y|^{n+2s} }\,dy =1. \end{aligned} \end{aligned}$$

$$\square $$

#### Remark 3.6

By requiring that $\Omega$ be compactly contained in $B_M$, one obtains a strict super-solution. However, we will not need this.

## Boundary Harnack inequality in 1D

We are interested in one-dimensional (1D) Dirichlet problems on the half space $\mathbb {R}^n_+$. Note that for $x=(x',x_n)\in \mathbb {R}^n_+$,

$$\begin{aligned}\begin{aligned} \mathcal {L}_{\mathbb {R}^n_+}u(x)&=C_{n,s} x_n^{2s-2} \text {P.V.}\,\int _{|y|<x_n} \dfrac{ u(x)- \frac{u(x+y)+u(x-y)}{2} }{|y|^{n+2s}} \,dy\\&=C_{n,s}|\mathbb {S}^{n-2}| \int _0^{x_n} \int _0^{\pi } \dfrac{ u(x',x_n) -\frac{ u(x'+y',x_n+r\cos \theta ) +u(x'+y',x_n-r\cos \theta ) }{2} }{ r^{1+2s} }\\ {}&\quad \sin ^{n-2}\theta \,d\theta \,dr. \end{aligned}\end{aligned}$$

Throughout this section we assume that *u* is 1D (i.e. *u* depends only on $x_n$). Then

$$\begin{aligned}&\mathcal {L}_{\mathbb {R}^n_+}u(x_n) =C_{n,s}|\mathbb {S}^{n-2}| \int _0^{x_n} \int _0^{\pi } \dfrac{ u(x_n) -\frac{ u(x_n+r\cos \theta ) +u(x_n-r\cos \theta ) }{2} }{ r^{1+2s} } \sin ^{n-2}\theta \,d\theta \,dr,\\&\qquad \forall x_n>0. \end{aligned}$$

It is convenient to write $x\in \mathbb {R}_+$ in place of $x_n$. In this section we will prove the following

### Proposition 1.15

(Boundary regularity in 1D) Suppose $u\in C^{2s+}((0,1))\cap C([0,2))$ is a 1D solution to

4.1 $$\begin{aligned} {\left\{ \begin{array}{ll} \mathcal {L}_{\mathbb {R}^n_+}u=0 &{} \text { on }(0,1),\\ u>0 &{} \text { on }(0,2),\\ u(0)=0. \end{array}\right. }\end{aligned}$$

There exists $\alpha _*\in (0,2\,s \wedge 1)$ and $C>0$ such that

$$\begin{aligned} \left\| { \dfrac{u}{x} }\right\| _{C^{\alpha _*}((0,\frac{1}{2}))} \le Cu(1). \end{aligned}$$

Here the *C* and $\alpha _*$ depend only on *n* and *s*.

### Remark 4.2

Note that the function *x* is a model solution to (4.1), and in fact the unique solution on $\mathbb {R}_+$ up to a constant multiple, as we will show in Lemma 6.2. In other words, any two solutions are comparable up to the boundary in a Hölder continuous way.

### Preliminaries

The scaling property Lemma A.4 allows us to compute the action of $\mathcal {L}_{\mathbb {R}_+}$ on monomials.

#### Lemma 1.17

(Monomials on the half line) For any $p\ge 0$,

$$\begin{aligned} \mathcal {L}_{\mathbb {R}^n_+}x^p =a(p)x^{p-2} \quad \text { on }\mathbb {R}_+, \end{aligned}$$

where $a(p)=-\psi (p,1)$, as defined in (B.1). In particular, $a(0)=a(1)=0$ and $a(2)=-2$.

#### Remark 4.4

When $n=1$, by a series expansion,

$$\begin{aligned} a'(p)&= -C_{1,s} \int _0^1 \dfrac{ (1+y)^p \log (1+y) +(1-y)^p \log (1-y) }{ y^{1+2s} } \,dy\\&=\sum _{k\ge 0,\,\ell \ge 1,\,k+\ell \ge 2} \left( {\begin{array}{c}p\\ k\end{array}}\right) \dfrac{1}{\ell } [(-1)^k+(-1)^\ell ] \int _0^1 \dfrac{ y^{k+\ell } }{ y^{1+2s} } \,dy\\&=\sum _{m=1}^{\infty } \sum _{\ell =1}^{2m} \dfrac{ (-1)^{\ell } }{ \ell (m-s) } \left( {\begin{array}{c}p\\ 2m-\ell \end{array}}\right) . \end{aligned}$$

However, it is not clear from this expression if *a*(*p*) is monotone or signed for large *p*.

#### Proof

Let $r>0$. Applying Lemma A.4 to $\Omega =r^{-1}\Omega =\mathbb {R}^n_+$ and $u(x)=x^p$, we see that

$$\begin{aligned}\begin{aligned} \mathcal {L}_{\mathbb {R}^n_+}(rx)^{p}\big |_{x=1} =r^2 \mathcal {L}_{\mathbb {R}^n_+}x^{p}\big |_{x=r}. \end{aligned}\end{aligned}$$

By linearity,

$$\begin{aligned}\begin{aligned} \mathcal {L}_{\mathbb {R}^n_+}x^p\big |_{x=r} =r^{p-2} \mathcal {L}_{\mathbb {R}^n_+}x^{p}\big |_{x=1}. \end{aligned}\end{aligned}$$

Thus $$a(p)=\mathcal {L}_{\mathbb {R}^n_+}x^p\big |_{x=1}$$. $$\square $$

We will use the following version of strong maximum principle for functions with non-negative data in the adjacent interval of the same length.

#### Lemma 1.19

(Strong maximum principle) Suppose $u\in C^{2s+}((0,1))\cap C([0,2))$ solves

4.2 $$\begin{aligned} {\left\{ \begin{array}{ll} \mathcal {L}_{\mathbb {R}^n_+}u\ge 0 &{} \text { in }(0,1),\\ u\ge 0 &{} \text { in }[1,2),\\ u(0)\ge 0. \end{array}\right. }\end{aligned}$$

Then either $u\equiv 0$ on (0, 1), or

$$\begin{aligned} u> 0 \quad \text { on }(0,1). \end{aligned}$$

#### Proof

This is simply Proposition 2.2 with $G=\mathbb {R}^{n-1}\times (0,1)$ and $G_*=\mathbb {R}^{n-1}\times (0,2)$. $\square$

### Boundary Harnack inequality

First of all we show Proposition 4.1 for $\alpha =0$, using interior Harnack inequality and comparison arguments.

#### Lemma 1.20

(Two-sided estimate) Suppose $u\in C^{2s+}((0,1))\cap C([0,2))$ solves

$$\begin{aligned}{\left\{ \begin{array}{ll} \mathcal {L}_{\mathbb {R}^n_+}u=0 &{} \text { in }(0,1),\\ u>0 &{} \text { in }(0,2),\\ u(0)=0, \end{array}\right. }\end{aligned}$$

then there exists $C>0$ universal such that

$$\begin{aligned} C^{-1}u(1) \le \dfrac{u(x)}{x} \le C u(1) \quad \text { on }(0,1]. \end{aligned}$$

#### Proof

By replacing *u* by *u*/*u*(1) if necessary, we may assume that $u(1)=1$. By Lemma 2.3, there exists $C>0$ universal such that

$$\begin{aligned} C^{-1} \le u(x) \le C \quad \text { for }x\in [\tfrac{1}{2},1]. \end{aligned}$$

Applying Lemma 4.5 to $u-C^{-1}x$ and $2Cx-u$ on $(0,\tfrac{1}{2})$ yields the result. $\square$

#### Corollary 1.21

(Boundary Harnack inequality) Let $u\in C^{2s+}((0,1))\cap C([0,2))$ be a solution to

4.3 $$\begin{aligned} {\left\{ \begin{array}{ll} \mathcal {L}_{\mathbb {R}^n_+}u=0 &{} \text { in }(0,1),\\ u>0 &{} \text { in }(0,2),\\ u(0)=0. \end{array}\right. }\end{aligned}$$

Then there exists $C>0$ universal such that

$$\begin{aligned} \sup _{x\in (0,1]} \dfrac{u(x)}{x} \le C \inf _{x\in (0,1]} \dfrac{u(x)}{x}. \end{aligned}$$

### Boundary Hölder regularity

#### Lemma 1.22

(Improvement of oscillation) Suppose $u\in C^{2s+}((0,1))\cap C([0,2))$ solves

$$\begin{aligned}{\left\{ \begin{array}{ll} \mathcal {L}_{\mathbb {R}^n_+}u=0 &{} \text { in }(0,1),\\ u>0 &{} \text { in }(0,2),\\ u(0)=0, \end{array}\right. }\end{aligned}$$

For $$k=1,2,\dots $$, denote

$$\begin{aligned} m_k=\inf _{x\in (0,4^{-k})}\frac{u(x)}{x}, \quad M_k=\sup _{x\in (0,4^{-k})}\frac{u(x)}{x}. \end{aligned}$$

Then there exists a universal constant $c\in (0,1)$ such that for any $k\ge 1$,

$$\begin{aligned} M_{k+1}-m_{k+1} \le c (M_k-m_k). \end{aligned}$$

#### Proof

By replacing *u* by *u*/*u*(1) if necessary, we may assume that $u(1)=1$. By Lemma 4.7, we can take $m_1=C_3^{-1}$ and $M_1=C_3$. We assume in the following that *u*(*x*)/*x* is not a constant; otherwise we can trivially take $M_k=m_k$ for all $k\ge 2$.

Suppose $M_k>m_k>0$ for $k\ge 1$ is known, such that

$$\begin{aligned} u - m_k x \ge 0 \quad \text { and }\quad M_k x - u \ge 0 \quad \text { in }(0,4^{-k}). \end{aligned}$$

By Lemma A.4, both the functions $(u-m_k)(2^{-1}4^{-k}x)$ and $(M_k-u)(2^{-1}4^{-k}x)$ are $\mathcal {L}_{\mathbb {R}_+}$-harmonic and non-negative on (0, 2). As they solve (4.2), the strong maximum principle Lemma 4.5, they are strictly positive on (0, 1). This means that

$$\begin{aligned} u - m_k x> 0 \quad \text { and }\quad M_k x - u > 0 \quad \text { in }(0,2^{-1}4^{-k}). \end{aligned}$$

Similarly, the functions $(u-m_k)(4^{-k-1}x)$ and $(M_k-u)(4^{-k-1}x)$ solve (4.3), so that Lemma 4.7 implies that

$$\begin{aligned}{\left\{ \begin{array}{ll} \displaystyle \sup _{x\in (0,1)} \dfrac{(u-m_k)(4^{-k-1}x)}{x} \le C \inf _{x\in (0,1)} \dfrac{(u-m_k)(4^{-k-1}x)}{x},\\ \displaystyle \sup _{x\in (0,1)} \dfrac{(M_k - u)(4^{-k-1}x)}{x} \le C \inf _{x\in (0,1)} \dfrac{(M_k - u)(4^{-k-1}x)}{x}. \end{array}\right. }\end{aligned}$$

Rescaling and multiplying throughout by the normalizing factor $4^{k+1}$, we have

$$\begin{aligned}{\left\{ \begin{array}{ll} \displaystyle \sup _{x\in (0,4^{-(k+1)})} \dfrac{u(x) - m_k x}{x} \le C \inf _{x\in (0,4^{-(k+1)})} \dfrac{u(x) - m_k x}{x},\\ \displaystyle \sup _{x\in (0,4^{-(k+1)})} \dfrac{M_k x - u(x)}{x} \le C \inf _{x\in (0,4^{-(k+1)})} \dfrac{M_k x - u(x)}{x}. \end{array}\right. }\end{aligned}$$

This means that

$$\begin{aligned}{\left\{ \begin{array}{ll} M_{k+1}-m_k \le C(m_{k+1}-m_k),\\ M_k-m_{k+1} \le C(M_k-M_{k+1}). \end{array}\right. }\end{aligned}$$

Adding up these two inequalities,

$$\begin{aligned} (M_{k+1}-m_{k+1})+(M_k-m_k) \le C\left( (M_k-m_k) -(M_{k+1}-m_{k+1}) \right) . \end{aligned}$$

Thus

$$\begin{aligned} M_{k+1}-m_{k+1} \le c(M_k-m_k), \quad c = \dfrac{C-1}{C+1}. \end{aligned}$$

Now a standard iteration yields the Hölder continuity of the quotient. $\square$

#### Proof of Proposition 4.1

By replacing *u* by *u*/*u*(1) if necessary, we assume that $u(1)=1$. By Lemma 2.4, we know that $u\in C^{\beta }((0,\tfrac{3}{4}))$ for some $\beta >0$ and $\left\| {u}\right\| _{C^{\beta }(B_{d/2}(d))} \le Cd^{-\beta }$ for $d>0$. Fix $\theta>(1+\beta )/\beta >1$. Let $x,y\in [0,1/4)$. Write $r=|x-y|$, $d=x\wedge y$. If $r\le d^{\theta }/2$, then by Lemma 2.4,

$$\begin{aligned} \left| \frac{u(x)}{x}-\frac{u(y)}{y}\right|&\le C\frac{1}{x} \left\| {u}\right\| _{C^{\beta }(B_{d/2}(d))} r^{\beta } +Cu(y) \left\| {\frac{1}{x}}\right\| _{C^{\beta }(B_{d/2}(d))} r^{\beta }\\&\le Cx^{-1}d^{-\beta }r^{\beta } +Cyd^{-1-\beta }r^{\beta } \le Cd^{-1-\beta }r^{\beta } \le Cr^{\beta -\frac{1+\beta }{\theta }}. \end{aligned}$$

If $r\ge \frac{d^\theta }{2}$, then $x,y\in (0,d+r)$ and by iterating Lemma 4.8, we have

$$\begin{aligned} \left| \frac{u(x)}{x}-\frac{u(y)}{y}\right| \le \sup _{(0,d+r)}\frac{u}{x} -\inf _{(0,d+r)}\frac{u}{x} \le C(d+r)^{\beta } \le Cr^{\frac{\beta }{\theta }}. \end{aligned}$$

Hence,

$$\begin{aligned} \left\| {\frac{u}{x}}\right\| _{C^{\alpha }([0,\frac{1}{4}))} \le C, \quad \text { for } \alpha =(\beta -\tfrac{1+\beta }{\theta }) \wedge \tfrac{\beta }{\theta }, \end{aligned}$$

as desired. $$\square $$

## Hölder regularity up to boundary

### Pointwise boundary Harnack inequality

Using the global maximum principle, we obtain a direct pointwise bound which is good for controlling the interior behavior.

#### Lemma 1.23

(Interior control) Let $u\in C^{2\,s+}(\Omega )\cap C({\overline{\Omega }})$ be a solution to

$$\begin{aligned}{\left\{ \begin{array}{ll} \mathcal {L}_\Omega u=f &{} \text { in }\Omega \\ u=g &{} \text { on }\partial \Omega . \end{array}\right. }\end{aligned}$$

Then

$$\begin{aligned} \left\| {u}\right\| _{L^\infty (\Omega )} \le \dfrac{({{\,\textrm{diam}\,}}\Omega )^2}{2n} \left\| {f}\right\| _{L^\infty (\Omega )} +\left\| {g}\right\| _{L^\infty (\partial \Omega )}. \end{aligned}$$

#### Proof

Use Lemma 2.1 on $\left\| {f}\right\| _{L^\infty (\Omega )}\varphi ^{(1)} +\left\| {g}\right\| _{L^\infty (\partial \Omega )} \pm u$, with $\varphi ^{(1)}$ given in Lemma 3.5. $\square$

We can now control a solution by the distance function.

#### Lemma 1.24

(Global boundary Harnack principle) Suppose $u\in C^{2\,s+}(\Omega )\cap C({\overline{\Omega }})$ solves

$$\begin{aligned}{\left\{ \begin{array}{ll} \mathcal {L}_\Omega u = f &{} \text { in }\Omega ,\\ u=0 &{} \text { on }\partial \Omega . \end{array}\right. }\end{aligned}$$

Then there exists a universal constant *C* such that

$$\begin{aligned} \left\| { \dfrac{u}{d} }\right\| _{L^\infty (\Omega )} \le C\left\| {f}\right\| _{L^\infty (\Omega )}. \end{aligned}$$

#### Proof

Since $\Omega$ is a bounded domain of class $C^{1,1}$, there exists $b>0$ such that an exterior tangent ball of radius *b* exists at each point $x_0\in \partial \Omega$. By Proposition 3.1, in a suitable coordinate system there exists $\varphi ^{(x_0)}$ such that

$$\begin{aligned}{\left\{ \begin{array}{ll} \mathcal {L}_{U}\bigl ( \left\| {f}\right\| _{L^\infty (\Omega )}\varphi ^{(x_0)} \pm u \bigr ) \ge 0 &{} \text { in }\Omega \cap B_{r_0}(x_0),\\ \left\| {f}\right\| _{L^\infty (\Omega )}\varphi ^{(x_0)} \pm u \ge 0 &{} \text { in }\bigl (\Omega \cap B_{2r_0}(x_0)\setminus B_{r_0}(x_0)\bigr ) \cup \partial \Omega . \end{array}\right. }\end{aligned}$$

Since

$$\begin{aligned} \bigcup _{x\in U \cap B_{r_0}(x_0)} B_{d_\Omega (x)}(x) \subset B_{2r_0}(x_0), \end{aligned}$$

Proposition 2.2 applies, we have

$$\begin{aligned} |u(x)|\le \left\| {f}\right\| _{L^\infty (\Omega )}\varphi ^{(x_0)}(x). \quad \forall x\in U \cap B_{r_0}(x_0) \end{aligned}$$

Since $\varphi ^{(x_0)}$ grows linearly away from the boundary, we have

$$\begin{aligned} |u(x)| \le C\left\| {f}\right\| _{L^\infty (\Omega )}d_\Omega (x), \quad \text { for }d_\Omega (x)<r_0. \end{aligned}$$

The interior estimate simply follows from Lemma 5.1. $\square$

We present a local analogue in a half ball $B_r^+=B_r \cap \left\{ x_n>0\right\}$, where $r>0$.

#### Lemma 1.25

(Local boundary Harnack principle) Suppose $u\in C^{2\,s+}(B_1^+) \cap C(\overline{B_2^+})$ solves

$$\begin{aligned}{\left\{ \begin{array}{ll} \mathcal {L}_{\mathbb {R}^n_+} u = 0 &{} \text { in }B_1^+,\\ u=0 &{} \text { on }\partial \mathbb {R}^n_+ \cap B_2^+. \end{array}\right. }\end{aligned}$$

Then

$$\begin{aligned} \left\| { \dfrac{u}{x_n} }\right\| _{L^\infty (B_{1/2}^+)} \le C\left\| {u}\right\| _{L^\infty (B_2^+)}. \end{aligned}$$

Here *C* depends only on *n* and *s*.

#### Proof

Let $x_0\in \partial \mathbb {R}^n_+\cap \partial B_1^+$. By Proposition 3.1 with $b=1$, there is a universal $r_0\in (0,1/2)$ such that

$$\begin{aligned}{\left\{ \begin{array}{ll} \mathcal {L}_{\mathbb {R}^n_+}\bigl ( \left\| {u}\right\| _{L^\infty (B_2^+)} \varphi ^{(x_0)} \pm u \bigr ) \ge 0, &{} \text { in }B_{r_0}^+(x_0),\\ \left\| {u}\right\| _{L^\infty (B_2^+)} \varphi ^{(x_0)} \pm u \ge 0 &{} \text { in }\bigl (B_{2r_0}^+(x_0) \setminus B_{r_0}^+(x_0)\bigr ) \cup \bigl ( \partial \mathbb {R}^n_+ \cap B_{r_0}(x_0) \bigr ). \end{array}\right. }\end{aligned}$$

By Proposition 2.2,

$$\begin{aligned} |u|\le \left\| {u}\right\| _{L^\infty (B_2^+)} \varphi ^{(x_0)} \quad \text { in }B_{r_0}^+(x_0). \end{aligned}$$

Now for each $x\in B_1^+ \cap \left\{ 0<x_n<r_0\right\}$ we choose $x_0=(x',0)$ to obtain

$$\begin{aligned} |u(x)|\le C\left\| {u}\right\| _{L^\infty (B_2^+)}x_n, \quad \text { in }\left\{ |x'|<1/2\right\} \times \left\{ 0<x_n<r_0\right\} . \end{aligned}$$

for *C* universal. The result follows by combining it with the trivial estimate

$$\begin{aligned} |u(x)|\le r_0^{-1}\left\| {u}\right\| _{L^\infty (B_2^+)}x_n \quad \text { in }\left\{ |x'|<1/2\right\} \times \left\{ r_0<x_n<1/2\right\} .\\ \end{aligned}$$

### Hölder regularity up to boundary

As above, we give a global and a local result. While practically having an order of 2*s* in the interior, the operator satisfies the classical Hopf boundary lemma. Thus the minimum of the two yields the combined regularity. This effect is analogously seen with the spectral fractional Laplacian.

#### Proposition 1.26

(Global boundary regularity) Suppose $u\in C^{2\,s+}(\Omega )\cap C({\overline{\Omega }})$ solves

$$\begin{aligned} {\left\{ \begin{array}{ll}{l} \mathcal {L}_\Omega u = f &{} \text { in }\Omega ,\\ u=0 &{} \text { in }\partial \Omega . \end{array}\right. }\end{aligned}$$

Then for any $\epsilon \in (0,1)$, there exists a constant $C=C(n,s,\Omega ,\epsilon )>0$ such that

$$\begin{aligned}{\left\{ \begin{array}{ll} \left\| {u}\right\| _{C^{0,1}({\overline{\Omega }})} \le C\left\| {f}\right\| _{L^\infty (\Omega )} &{} \text { for }s\in (\frac{1}{2},1),\\ \left\| {u}\right\| _{C^{1-\varepsilon }({\overline{\Omega }})} \le C\left\| {f}\right\| _{L^\infty (\Omega )} &{} \text { for }s=\frac{1}{2},\\ \left\| {u}\right\| _{C^{2s}({\overline{\Omega }})} \le C\left\| {f}\right\| _{L^\infty (\Omega )} &{} \text { for }s\in (0,\frac{1}{2}).\\ \end{array}\right. }\end{aligned}$$

#### Proof

By dividing by $\left\| {f}\right\| _{L^\infty (\Omega )}$ if necessary, we can assume $\left\| {f}\right\| _{L^\infty (\Omega )}\le 1.$ By Lemma 5.2,

5.1 $$\begin{aligned} |u|\le C d \quad \text { in }\Omega . \end{aligned}$$

Let

$$\begin{aligned} \beta ={\left\{ \begin{array}{ll} 1 &{} \text { for }s\in (\frac{1}{2},1),\\ 1-\epsilon &{} \text { for }s=\frac{1}{2},\\ 2s &{} \text { for }s\in (0,\frac{1}{2}). \end{array}\right. }\end{aligned}$$

We need to show that

$$\begin{aligned} |u(x)-u(y)| \le C |x-y|^\beta \quad \forall x,y\in \overline{\Omega }. \end{aligned}$$

Write $\rho =\min \left\{ d(x),d(y)\right\} =d(x)$, by interchanging *x* and *y* if necessary.

Case 1: $4|x-y|<\rho$. Then $y\in B_{\rho /4}(x)\subset B_\rho (x)\subset \Omega$. By Lemma A.3 and Lemma A.4, the rescaled function $u_\rho (z)=u(x+\rho z)$ satisfies
  5.2 $$\begin{aligned} \mathcal {L}_{\rho ^{-1}(\Omega -x)} u_\rho (z) =f_\rho (z):=\rho ^2 f(x+\rho z) \quad \text { in }B_{1/4}\subset B_1\subset \Omega . \end{aligned}$$
  Using the interior estimates Lemma 2.4, we have
  $$\begin{aligned}{} & {} \left\| {u_\rho }\right\| _{C^{\beta }(B_{1/4})} \le C\left( \left\| {u_\rho }\right\| _{L^\infty (B_1)} +\left\| {u_\rho }\right\| _{L^1_{2s}(\Omega )}\right. \\{} & {} \left. +\left\| { d_{ \rho ^{-1}(\Omega -x_0) } }\right\| _{L^\infty (B_1)}^{2-2s} \left\| {f_\rho }\right\| _{L^\infty (B_1)} \right) \end{aligned}$$
  In view of (5.1) we observe that
  $$\begin{aligned} \begin{aligned} \left[ {u_\rho }\right] _{C^{\beta }(B_{1/4})}&= \rho ^\beta \left[ {u}\right] _{C^{\beta }(B_{\rho /4}(x))}\\ \left\| {u_\rho }\right\| _{L^\infty (B_1)}&\le C \left\| {d}\right\| _{L^\infty (B_\rho (x))} \le C \rho \le C\rho ^\beta \\ \end{aligned}\\ \begin{aligned}&\left\| {u_\rho }\right\| _{L^1_{2s}(\Omega )} = \left\| {u(x+\rho \cdot )}\right\| _{L^1_{2s}(\Omega )}\\&\le C\left\| {d(x+\rho \cdot )}\right\| _{L^1_{2s}(\Omega )}\\&\le Cd(x)\left\| {1}\right\| _{L^1_{2s}(\Omega )} +C\rho \left\| {1}\right\| _{L^1_{2s-1}(\Omega )}\\&\le C\rho \left( 1+\int _{ 1\le |z| \le \rho ^{-1} {{\,\textrm{diam}\,}}\Omega } \dfrac{1}{|z|^{n+2s-1}} \,dz \right) \\&\le {\left\{ \begin{array}{ll} C\rho &{} \text { for }s\in (\frac{1}{2},1),\\ C\rho (1+\log \frac{1}{\rho }) &{} \text { for }s=\frac{1}{2},\\ C\rho (1+\rho ^{2s-1}) &{} \text { for }s\in (0,\frac{1}{2}),\\ \end{array}\right. }\\&\le C\rho ^\beta . \end{aligned}\\ \begin{aligned}&\left\| { d_{ \rho ^{-1}(\Omega -x_0) } }\right\| _{L^\infty (B_1)}^{2-2s} \left\| {f_\rho }\right\| _{L^\infty (B_1)}\\&\le \left\| {d}\right\| _{L^\infty (\Omega )}^{2-2s}\rho ^{2s-2} \cdot \rho ^2 \le C \rho ^{2s} \le C \rho ^\beta . \end{aligned}\end{aligned}$$
  We conclude that
  $$\begin{aligned} \left[ {u}\right] _{C^{\beta }(B_{\rho /4}(x))} \le C \quad \textit{ i.e. } \quad |u(x)-u(y)|\le C |x-y| \quad \text { for }|x-y|<\frac{\rho }{4}. \end{aligned}$$
Case 2: $$|x-y|\ge \frac{\rho }{4}$$. Then
  $$\begin{aligned}\begin{aligned} |u(x)-u(y)| \le |u(x)|+|u(y)|&\le C \left( d(x)+d(y)\right) \\&\le C (2d(x)+|x-y|)\\&\le C |x-y| \le C|x-y|^\beta . \end{aligned}\end{aligned}$$

$$\square $$

#### Proposition 1.27

(Local boundary regularity) Suppose $u\in C^{2\,s+\varepsilon }_{\text {loc}}(B_1^+)\cap C(\overline{B_2^+})$ solves

$$\begin{aligned} {\left\{ \begin{array}{ll} \mathcal {L}_{\mathbb {R}^n_+}u = 0 &{} \text { in }B_1^+,\\ u=0 &{} \text { in }\partial \mathbb {R}^n_+\cap B_2. \end{array}\right. }\end{aligned}$$

Then for any $\epsilon \in (0,1)$, there exists a constant $C=C(n,s,\epsilon )>0$ such that

$$\begin{aligned}{\left\{ \begin{array}{ll} \left\| {u}\right\| _{C^{0,1}(\overline{B_{1/16}^+})} \le C\left\| {u}\right\| _{L^\infty (B_2^+)} &{} \text { for }s\in (\frac{1}{2},1),\\ \left\| {u}\right\| _{C^{1-\epsilon }(\overline{B_{1/16}^+})} \le C\left\| {u}\right\| _{L^\infty (B_2^+)} &{} \text { for }s=\frac{1}{2},\\ \left\| {u}\right\| _{C^{2s}(\overline{B_{1/16}^+})} \le C\left\| {u}\right\| _{L^\infty (B_2^+)} &{} \text { for }s\in (0,\frac{1}{2}).\\ \end{array}\right. }\end{aligned}$$

#### Proof

By normalizing if necessary, we assume $\left\| {u}\right\| _{L^\infty (B_2^+)}\le 1$. LetWe need to show that

$$\begin{aligned} |u(x)-u(y)| \le C |x-y|^\beta \quad \forall x,y\in \overline{B_{1/16}^+}. \end{aligned}$$

Without loss of generality let $\rho =x_n\le y_n$. By Lemma 5.3 we have

5.3 $$\begin{aligned} |u| \le C x_n \quad \text { in }\overline{B_{1/2}^+}. \end{aligned}$$

**Case 1:** $4|x-y|<\rho$. Then $y\in B_{\rho /4}(x)\subset B_\rho (x)\subset B_1^+$. As in the proof of Proposition 5.4, the rescaled function $u_\rho (z)=u(x+\rho z)$ they satisfy the equation (note $\rho ^{-1}\ge 16$)

$$\begin{aligned} \mathcal {L}_{\rho ^{-1}(B_2^+-x)}u_\rho =0 \quad \text { in }B_1\subset B_4\subset \rho ^{-1}(B_1^+-x). \end{aligned}$$

By (5.3),

$$\begin{aligned} |u_\rho (z)| \le C (x_n+\rho z_n) \quad \text { in }\rho ^{-1}(B_{1/2}^+-x). \end{aligned}$$

From Lemma 2.4 we have the estimate

$$\begin{aligned} \left\| {u_\rho }\right\| _{C^{\beta }(B_{1/4})} \le C\left( \left\| {u_\rho }\right\| _{L^\infty (B_1)} +\left\| {u_\rho }\right\| _{L^1_{2s}(\rho ^{-1}(B_1^+-x))} \right) . \end{aligned}$$

Therefore, by (5.3),

$$\begin{aligned}\begin{aligned} \rho ^{\beta } \left[ {u}\right] _{C^{\beta }(B_{\rho /4}(x))}&\le C\left( \left\| {u}\right\| _{L^\infty (B_\rho (x))} +\left\| {x_n+\rho z_n}\right\| _{L^1_{2s}(\rho ^{-1}(B_2^+-x))} \right) \\&\le C\left( x_n +x_n\left\| {1}\right\| _{L^1_{2s}(\mathbb {R}^{n-1})} +\rho \left\| {1}\right\| _{L^1_{2s-1}(\rho ^{-1}B_4)} \right) \\&\le C\rho ^\beta . \end{aligned}\end{aligned}$$

**Case 2:** $4|x-y|\ge \rho$. Then by (5.3),

$$\begin{aligned}\begin{aligned} |u(x)-u(y)| \le x_n+y_n \le 2\rho +|y_n-x_n| \le 9|x-y| \le C|x-y|^\beta .\\ \end{aligned}\end{aligned}$$

$$\square $$

## Liouville-type results

In this section we classify solutions to homoegenous Dirichlet problems in a half space with controlled growth. We write $\mathbb {R}^n_+=\left\{ x=(x',x_n)\in \mathbb {R}^{n-1}\times \mathbb {R}_+\right\}$.

### Proposition 1.28

(Liouville-type result) Let *v* be a solution to

6.1 $$\begin{aligned} {\left\{ \begin{array}{ll} \mathcal {L}_{\mathbb {R}^n_+}v=0 &{} \text { in }\mathbb {R}^n_+,\\ v=0 &{} \text { on }\partial \mathbb {R}^n_+, \end{array}\right. }\end{aligned}$$

which satisfies the growth condition

6.2 $$\begin{aligned} |v(x)|\le C(1+|x|^{1+\alpha }), \end{aligned}$$

for some $\alpha \in (0,\alpha _*)$ with $\alpha _*\in (0,2s\wedge 1)$ given in Proposition 4.1. Then *v* is a 1D and linear, i.e.

$$\begin{aligned} v(x)=b_0x_n, \end{aligned}$$

for some constant $b_0\in \mathbb {R}$.

### Lemma 1.29

(Liouville in 1D) If ${\bar{v}}$ solves

$$\begin{aligned}{\left\{ \begin{array}{ll} \mathcal {L}_{\mathbb {R}_+}{\bar{v}}=0 &{} \text { in }(0,+\infty )\\ {\bar{v}}(0)=0, \end{array}\right. }\end{aligned}$$

and satisfies the growth condition

$$\begin{aligned} |{\bar{v}}(x)| \le C(1+|x|^{1+\alpha }), \end{aligned}$$

where $\alpha \in (0,\alpha _*)$ with $\alpha _*\in (0,2s\wedge 1)$ given in Proposition 4.1. Then

$$\begin{aligned} {\bar{v}}(x)=c_0 x, \end{aligned}$$

for some $$c_0\in \mathbb {R}$$.

### Proof

Let

$$\begin{aligned} {\bar{v}}_R(x)=R^{-1-\alpha _*}{\bar{v}}(R x), \end{aligned}$$

which satisfies the growth condition

$$\begin{aligned} |{\bar{v}}_R(x)| \le CR^{-1-\alpha _*}(1+R^{1+\alpha }|x|^{1+\alpha }) \le CR^{-(\alpha _*-\alpha )}(1+|x|^{1+\alpha }). \end{aligned}$$

In particular,

$$\begin{aligned} \left\| {{\bar{v}}_R}\right\| _{L^\infty (0,2)} \le CR^{-(\alpha _*-\alpha )}. \end{aligned}$$

Applying Proposition 4.1 to ${\bar{v}}_R$, we see that

$$\begin{aligned} \left[ {\dfrac{{\bar{v}}}{x}}\right] _{C^{\alpha _*}(0,R)} =\left[ {\dfrac{{\bar{v}}_R}{x}}\right] _{C^{\alpha _*}(0,1)} \le C\left\| {{\bar{v}}_R}\right\| _{L^\infty (0,2)} \le CR^{-(\alpha _*-\alpha )} \rightarrow 0, \end{aligned}$$

as $R\rightarrow +\infty$. Hence ${\bar{v}}/x$ is a constant $c_0\in \mathbb {R}$. $\square$

### Lemma 1.30

(Solutions with slow growth vanish) Suppose *v* solves (6.1) with

$$\begin{aligned} |v(x)|\le C(1+|x|^{\beta }), \end{aligned}$$

for $$\beta \in [0,\beta _0)$$ where

6.3 $$\begin{aligned} \beta _0={\left\{ \begin{array}{ll} 1 &{} \text { for }s\in (1/2,1),\\ 1-\varepsilon &{} \text { for }s=1/2,\\ 2s &{} \text { for }s\in (0,1/2). \end{array}\right. }\end{aligned}$$

Then $$v\equiv 0$$.

### Proof

The rescaled function $v_R(x)=R^{-\beta _0}v(Rx)$ satisfies (6.1) and the growth condition

$$\begin{aligned} |v_R(x)| \le CR^{-\beta _0}(1+R^\beta |x|^\beta ) \le CR^{-(\beta _0-\beta )}(1+|x|^\beta ). \end{aligned}$$

By Proposition 5.5,

$$\begin{aligned} \left[ {v}\right] _{C^{\beta _0}(B_{R/16}^+)} =\left[ {v_R}\right] _{C^{\beta _0}(B_{1/16}^+)} \le C\left\| {v_R}\right\| _{L^\infty (B_2^+)} \le CR^{-(\beta _0-\beta )} \rightarrow 0, \end{aligned}$$

as $$R\rightarrow \infty $$. Hence, $$v\equiv v(0)=0$$. $$\square $$

### Lemma 1.31

(Solutions with mild growth are 1D) Suppose *v* satisfies (6.1) and the growth condition

$$\begin{aligned} |v(x)|\le C(1+|x|^{\beta }) \end{aligned}$$

for $\beta \in [\beta _0,2\beta _0)\cap (0,1+\alpha _*)$ where $\beta _0$ is as in (6.3) and $\alpha _* \in (0,2s\wedge 1)$ is given in Proposition 4.1. Then *v* is a 1D, i.e.

$$\begin{aligned} v(x)=b_0 x_n, \end{aligned}$$

for some $$b_0\in \mathbb {R}$$.

### Proof

Let $h\in (0,1]$ and $\omega \in \mathbb {S}^{n-1}\cap \left\{ x_n=0\right\}$. Write

$$\begin{aligned} w(x) =\dfrac{ v(x+h\omega )-v(x) }{ h^{\beta _0} }, \end{aligned}$$

which satisfies (6.1) and the growth condition (via the rescaling as in Lemma 6.3)

$$\begin{aligned} \left\| {w}\right\| _{L^\infty (B_{R/32})} \le \left[ {v}\right] _{C^{\beta _0}(B_{R/16})} \le CR^{-\beta _0}\left\| {v}\right\| _{L^\infty (B_{2R})} \le R^{\beta -\beta _0}. \end{aligned}$$

Since $\beta -\beta _0\in [0,\beta _0)$, Lemma 6.3 implies $w\equiv 0$. Then $v(x+h\omega )=v(x)$ for any $x\in \mathbb {R}^n_+$, $h\in (0,1]$, $\omega \in \mathbb {S}^{n-1}\cap \left\{ x_n=0\right\}$. Since $(h\omega )\mathbb {Z}$ is arbitrary on $\left\{ x_n=0\right\}$, *v* depends only on $x_n$. By Lemma 6.2, $v(x)=b_0x_n$ for some $b_0\in \mathbb {R}$. $\square$

### Proof of Proposition 6.1

We will prove by induction in *k* the following claim: if *v* satisfies (6.1) and the growth condition

6.4 $$\begin{aligned} |v(x)|\le C(1+|x|^{k\beta _0}) \end{aligned}$$

and $k\beta _0<1+\alpha _*$, then *v* is 1D and linear.

By Lemma 6.4, this is true for $k=1$. Suppose the claim is true for *k* and *v* is a solution to (6.1) satisfying

$$\begin{aligned} |v(x)|\le C(1+|x|^{(k+1)\beta _0}). \end{aligned}$$

By the rescaling argument and boundary regularity (e.g. in Lemma 6.4), the Hölder difference quotient $\frac{v(x+h\omega )-v(x)}{h^{\beta _0}}$ satisfies (6.1) and (6.4). Hence, there exists $b_0(h,\omega )$ such that

6.5 $$\begin{aligned} v(x+h\omega )-v(x)=b_0(h,\omega )x_n. \end{aligned}$$

By iterating (6.5) for $h=1$, we have

$$\begin{aligned} v(x+R\omega )-v(x)=b_0(1,\omega ) Rx_n. \end{aligned}$$

for any $R\in {\mathbb {N}}$. But then for $x=(0,R)$, by (6.4) (recall that $(k+1)\beta _0<1+\alpha _*<2$) we have

$$\begin{aligned} |v(R\omega ,R)| =|v(0,R)+b_0(1,\omega )R^2| \ge |b_0(1,\omega )|R^2 -CR^{1+\alpha } \ge \dfrac{|b_0(1,\omega )|}{2} R^2, \end{aligned}$$

contradicting (6.4) unless $b_0(1,\omega )\equiv 0$ for all $\omega \in \mathbb {S}^{n-1}\cap \left\{ x_n=0\right\}$. In view of (6.5), *v* depends only on $x_n$ and the result follows from Lemma 6.2. $\square$

## Proof of the higher regularity

### Proof of Theorem 1.1

In view of the interior estimates in Lemma 2.4, we just need to prove the following expansion: for some $\alpha =\alpha (n,s)\in (0,1)$ so small that Proposition 6.1 holds, for any $z\in \partial \Omega$, there exists $Q_z\in \mathbb {R}$, $r>0$ such that for any $x\in \Omega \cap B_r(z)$,

7.1 $$\begin{aligned} |u(x)-Q_z d(x)|\le C|x-z|^{1+\alpha }. \end{aligned}$$

Indeed, as in the proof of Proposition 4.1, one can interpolate the (degenerate) interior estimate with (7.1) to obtain the full $C^{1,\alpha '}(\overline{\Omega })$ regularity (for some $\alpha '\in (0,\alpha )$).

Suppose on the contrary that there exists $z\in \partial \Omega$ such that (7.1) does not hold for any $Q\in \mathbb {R}$, i.e.

$$\begin{aligned} \sup _{r\in (0,1]} r^{-1-\alpha } \left\| {u-Qd}\right\| _{L^\infty (B_r(z))} =\infty , \quad \forall Q\in \mathbb {R}. \end{aligned}$$

We split the proof by contradiction into a number of steps.

*Step 1: Choosing one* *Q* *for each* *r*.

For each $r>0$ small, we choose a *Q*(*r*) that minimizers $\left\| {u-Qd}\right\| _{L^2(B_r(z))}$, i.e.

$$\begin{aligned} Q(r)=\dfrac{ \int _{B_r(z)}ud\,dx }{ \int _{B_r(z)}d^2\,dx }. \end{aligned}$$

We claim that

7.2 $$\begin{aligned} \sup _{r\in (0,1]} r^{-1-\alpha } \left\| {u-Q(r)d}\right\| _{L^\infty (B_r(z))} =\infty . \end{aligned}$$

Suppose on the contrary that (7.2) does not hold, i.e there exists a (large) ${\bar{C}}>0$ such that

$$\begin{aligned} \left\| {u-Q(r)d}\right\| _{L^\infty (B_r(z))} \le {\bar{C}} r^{1+\alpha } \quad \forall r\in (0,1]. \end{aligned}$$

Then, for any $x\in B_r(z)$,

$$\begin{aligned} |Q(2r)-Q(r)|d(x) \le |u(x)-Q(2r)d(x)|+|u(x)-Q(r)d(x)| \le 2{\bar{C}} r^{1+\alpha }. \end{aligned}$$

Since $$\sup _{B_r(z)}d=r$$,

$$\begin{aligned} |Q(2r)-Q(r)|\le 2{\bar{C}} r^\alpha . \end{aligned}$$

Since for any $j\ge i \ge 0$,

$$\begin{aligned} \left| Q(2^{-i}r)-Q(2^{-j}r)\right| \le {\bar{C}} r^\alpha \sum _{k=i}^{j-1}2^{-k\alpha } \le C {\bar{C}} 2^{-i\alpha }r^\alpha , \end{aligned}$$

the limit $Q_0:=\lim _{r\searrow 0}Q(r)$ exists, and by fixing $i=0$ and letting $j\rightarrow \infty$,

$$\begin{aligned} |Q_0-Q(r)|\le C{\bar{C}} r^{\alpha }. \end{aligned}$$

In particular, putting $r=1$ implies $|Q_0|\le C({\bar{C}}+1)$, since $|Q(1)|\le C$. Hence, for all $r\in (0,1]$,

$$\begin{aligned}\begin{aligned} \left\| {u-Q_0d}\right\| _{L^\infty (B_r(z))}&\le \left\| {u-Q(r)d}\right\| _{L^\infty (B_r(z))} +\left\| {(Q_0-Q(r))d}\right\| _{L^\infty (B_r(z))}\\&\le C{\bar{C}} r^{1+\alpha } +C{\bar{C}} r^\alpha \sup _{B_r(z)}d \le Cr^{1+\alpha }, \end{aligned}\end{aligned}$$

a contradiction. This proves (7.2).

*Step 2: The blow-up sequence and growth bound.*

Now we define the monotone quantity

$$\begin{aligned} \theta (r):= \max _{{\bar{r}}\in [r,1]} ({\bar{r}})^{-1-\alpha } \left\| {u-Q({\bar{r}})d}\right\| _{L^\infty (B_{{\bar{r}}}(z))}. \end{aligned}$$

From $\lim _{r\searrow 0}\theta (r)=\infty$, there is a sequence $r_m\rightarrow 0$ such that

$$\begin{aligned} (r_m)^{-1-\alpha } \left\| {u-Q(r_m)d}\right\| _{L^\infty (B_{r_m}(z))} =\theta (r_m)\rightarrow \infty . \end{aligned}$$

Define the blow-up sequence $v_m:(r_m)^{-1}(\Omega -z)\rightarrow \mathbb {R}$,

$$\begin{aligned} v_m(x):=\dfrac{ u(z+r_m x)-Q(r_m)d(z+r_m x) }{ (r_m)^{1+\alpha }\theta (r_m) }, \end{aligned}$$

which satisfies

7.3 $$\begin{aligned} \left\| {v_m}\right\| _{L^\infty (B_1)}=1 \end{aligned}$$

and, from the choice of $Q(r_m)$,

7.4 $$\begin{aligned} \int _{B_1} v_m(x)d(z+r_m x)\,dx=0. \end{aligned}$$

We claim the following growth control

7.5 $$\begin{aligned} \left\| {v_m}\right\| _{L^\infty (B_R \cap (r_m)^{-1}(\Omega -z))} \le CR^{1+\alpha } \quad \forall R\ge 1. \end{aligned}$$

Indeed, the arguments in *Step 1* (replacing $\theta$ by $\theta (r)$, and the interval (0, 1] by [*r*, 1]) shows that

$$\begin{aligned} |Q(Rr)-Q(r)|\le C (Rr)^{\alpha } \theta (r) \quad \forall R\ge 1. \end{aligned}$$

Also since $\theta$ is non-increasing,

$$\begin{aligned} \theta (R r_m)\le \theta (r_m). \end{aligned}$$

Then (here we implicitly extend suitable functions by 0 outside $\Omega$)

$$\begin{aligned}\begin{aligned}&\left\| {v_m}\right\| _{L^\infty (B_R)} = \dfrac{1}{(r_m)^{1+\alpha }\theta (r_m)} \left\| {u-Q(r_m)d}\right\| _{L^\infty (B_{R r_m}(z))}\\&\quad \le \dfrac{1}{(r_m)^{1+\alpha }\theta (r_m)} \left( \left\| {u-Q(R r_m)d}\right\| _{L^\infty (B_{R r_m}(z))} +\left| Q(R r_m)-Q(r_m)\right| (R r_m) \right) \\&\quad \le \dfrac{1}{(r_m)^{1+\alpha }\theta (r_m)} (R r_m)^{1+\alpha }\theta (R r_m) +\dfrac{C}{(r_m)^{1+\alpha }\theta (r_m)} (R r_m)^{\alpha } \theta (r_m) \cdot (R r_m)\\&\quad \le R^{1+\alpha } + CR^{1+\alpha }. \end{aligned}\end{aligned}$$

This proves (7.5).

*Step 3: Equation for the blow-up sequence.*

Let $\Omega _m=(r_m)^{-1}(\Omega -z)$, which converges to a halfspace $\left\{ x\cdot e>0\right\}$ as $m\rightarrow +\infty$, for $e=-\nu (z)$, the inward normal at $z\in \partial \Omega$. By the properties in Lemma A.3 and Lemma A.4, the functions $v_m$ satisfy

7.6 $$\begin{aligned} \begin{aligned} |\mathcal {L}_{\Omega _m}v_m(x)|&= \dfrac{ 1 }{ (r_m)^{1+\alpha }\theta (r_m) } \left| \mathcal {L}_{(r_m)^{-1}(\Omega -z)}u(x) -Q(r_m)\mathcal {L}_{(r_m)^{-1}(\Omega -z)}d(x) \right| \\&= \dfrac{ (r_m)^2 }{ (r_m)^{1+\alpha }\theta (r_m) } \left| \mathcal {L}_{\Omega }u(z+r_m x) -Q(r_m)\mathcal {L}_{\Omega }d(z+r_m x) \right| \rightarrow 0, \end{aligned}\end{aligned}$$

since $\mathcal {L}_\Omega u$ and $\mathcal {L}_\Omega d=\mathcal {L}_{\Omega }\delta$ are bounded in view of Lemma A.2. Now, by Proposition 5.4, $\left\| {v_m}\right\| _{C^{2\beta }(\Omega _m)}\le C$ for some $\beta >0$. So Arzelà–Ascoli Theorem asserts a subsequence of $v_m$ uniformly converging on compact sets in $\left\{ x\cdot e>0\right\}$ to some function $v\in C^\beta (\left\{ x\cdot e>0\right\} )$, $\beta \in (0,1)$. Passing to the limit in (7.5) and (7.6) yields

$$\begin{aligned} \left\| {v}\right\| _{L^\infty (B_R \cap \left\{ x\cdot e>0\right\} )} \le CR^{1+\alpha } \quad \forall R\ge 1. \end{aligned}$$

and

$$\begin{aligned}{\left\{ \begin{array}{ll} \mathcal {L}_{\left\{ x\cdot e>0\right\} }v=0 &{} \text { in }\left\{ x\cdot e>0\right\} \\ v=0 &{} \text { on }\left\{ x\cdot e=0\right\} . \end{array}\right. }\end{aligned}$$

*Step 4: Classification of the limit, and the contradiction. *

By Proposition 6.1, $v(x)=c_0(x\cdot e)$ for some constant $c_0\in \mathbb {R}$. Using the fact that

$$\begin{aligned} \dfrac{d(z+r_m x)}{r_m} \rightarrow x\cdot e \quad \text { as }m\rightarrow +\infty , \end{aligned}$$

we pass to the limit in (7.4) (upon dividing by $r_m$) to see that

$$\begin{aligned} 0=\int _{B_1\cap \left\{ x\cdot e>0\right\} } v(x)(x\cdot e) \,dx =\int _{B_1\cap \left\{ x\cdot e>0\right\} } c_0(x\cdot e)^2 \,dx. \end{aligned}$$

But this implies $c_0=0$ and hence $v=0$, contradicting (7.3) in the limit $m\rightarrow +\infty$. Therefore (7.1) holds and the proof is complete. $\square$

## Existence of viscosity solution

Consider the Dirichlet problem

8.1 $$\begin{aligned} {\left\{ \begin{array}{ll} \mathcal {L}_{\Omega }u=f &{} \text { in }\Omega ,\\ u=g &{} \text { on }\partial \Omega . \end{array}\right. }\end{aligned}$$

We will establish the existence of a continuous viscosity solution using Perron’s method, carefully exploiting the mid-range maximum principle that $\mathcal {L}_{\Omega }$ satisfies. Throughout the section we assume $f\in C^{\alpha }(\Omega )$ and $g\in C(\partial \Omega )$, for some $\alpha >0$.

Our goal is to prove the following.

### Proposition 1.32

Let $\Omega \subset \mathbb {R}^n$ be a bounded domain of class $C^{1,1}$. Let $s\in (0,1)$ and $f\in C^\alpha$ for some $\alpha >0$. For any $f\in C^\alpha (\Omega )$ and $g\in C(\partial \Omega )$, There exists a unique $u\in C(\overline{\Omega })$ satisfying (8.1) in the viscosity sense. Moreover, $u\in C^{2\,s+\alpha }(\Omega )\cap C(\overline{\Omega })$ is a classical solution to (8.1).

The notion of viscosity solutions has been successfully used in nonlocal equations, see for example [1, 7, 11, 13]. For the proof, we extend the clean arguments described in [4] using the barrier constructed in Sect. 3.

### Definition 1.33

(Semi-continuous functions) We denote by

$$\begin{aligned} USC(\overline{\Omega }) \qquad \text {(resp. } LSC(\overline{\Omega }) \text {)} \end{aligned}$$

the space of upper (resp. lower) semi-continuous functions in $\overline{\Omega }$. For $u\in L^\infty (\overline{\Omega })$ we define the USC (resp. LSC) envelope as

$$\begin{aligned} u^*(x)=\sup _{x_k\rightarrow x} \limsup _{k\rightarrow \infty } u(x_k) \qquad \text {(resp. } u_*(x)=\inf _{x_k\rightarrow x} \liminf _{k\rightarrow \infty } u(x_k) \text {)}. \end{aligned}$$

We also need a localized definition based on Proposition 2.2.

### Definition 1.34

(Viscosity solutions) Let *G* be an non-empty, open set in $\Omega$. The domain of interaction of *G* is

$$\begin{aligned} G_*=\bigcup _{y\in G} B_{d_{\Omega }(y)}(y). \end{aligned}$$

(Thus, $\mathcal {L}_{\Omega }=\mathcal {L}_{G_*}$ in *G*.) Let $f\in C(\Omega )$. We say that $u\in USC(\overline{G_*})$ (resp. $u\in LSC(\overline{G_*})$) is a (mid-range) viscosity sub-solution of

$$\begin{aligned} \mathcal {L}_{G_*}u=f \text { in } G, \end{aligned}$$

if for any $x\in \Omega$, any neighborhood $N_x$ of *x* in $\Omega$ and any $\varphi \in C^2(N_x)\cap L^1(\overline{G_*})$ with

$$\begin{aligned} u(x)=\varphi (x), \quad u\le \varphi \, (\text {resp.} u\ge \varphi ) \text { in } \overline{G_*}, \end{aligned}$$

we have

$$\begin{aligned} \mathcal {L}_{G_*}\varphi (x_0) \le f(x_0) \qquad \text {(resp. } \mathcal {L}_{G_*}\varphi (x_0) \ge f(x_0) \text {).} \end{aligned}$$

In particular, when $G=\Omega$, $\overline{G_*}=\overline{\Omega }$. We say that $u\in C(\overline{\Omega })$ is a (global) viscosity solution in $\Omega$ if it is both a sub-solution and a super-solution in $\Omega$.

### Remark 8.4

Global sub-(resp. super-) solutions are necessarily mid-range sub-(resp. super-) solutions (but not vice versa). This is because the test function $\varphi \in L^1(\overline{\Omega })$ can be extended to keep the sign of $u-\varphi$ without affecting the computation of $\mathcal {L}_{\Omega }\varphi$ at the contact point.

### Comparison principle for viscosity solutions

We generalize Proposition 2.2 to viscosity solutions.

#### Lemma 1.36

(Mid-range maximum principle) Let $G\subseteq \Omega$ be open. Suppose $u\in LSC(\overline{G_*})$ solves, in the viscosity sense,

$$\begin{aligned}{\left\{ \begin{array}{ll} \mathcal {L}_{G_*} u \ge 0 &{} \text { in } G,\\ u\ge 0 &{} \text { in } \overline{G_*}\setminus G. \end{array}\right. }\end{aligned}$$

Then $$u\ge 0$$ in *G*.

#### Proof

If not, $\min _{\overline{\Omega }}u=-\delta$ for some $\delta >0$. Using a translated coordinate system if necessary, we assume that $0\in G$. The convex paraboloid

$$\begin{aligned} {\tilde{\varphi }}(x) = -\dfrac{\delta }{2} +\dfrac{\delta }{4(1+{{\,\textrm{diam}\,}}(G_*)^2)}|x|^2 \end{aligned}$$

takes values in $[-\delta /2,-\delta /4]$ and so (by moving down then up) there exists $c>0$ such that

$$\begin{aligned} \varphi (x)={\tilde{\varphi }}(x)-c \end{aligned}$$

touches *u* from below at some $x_0\in G$, i.e. $u(x_0)=\varphi (x_0)$ and $u\ge \varphi$ in *G*. By construction $u\ge 0\ge {{\tilde{\varphi }}}\ge \varphi$ in $\overline{G_*}{\setminus } G$. On the one hand, by Definition 8.3,

$$\begin{aligned} \mathcal {L}_{G_*}\varphi (x_0) \ge 0. \end{aligned}$$

On the other hand,

$$\begin{aligned} \mathcal {L}_{G_*}\varphi (x_0)&=\frac{C_{n,s}}{2} \frac{\delta }{4(1+{{\,\textrm{diam}\,}}(G_*)^2)} d_{U}(x_0)^{2s-2} \int _{|y|<d_{U}(x_0)} \frac{ 2|x|^2-|x+y|^2-|x-y|^2 }{ |y|^{n+2s} } \,dy\\&=-\frac{C_{n,s}}{2} \frac{\delta }{2(1+{{\,\textrm{diam}\,}}(G_*)^2)} d_{U}(x_0)^{2s-2} \int _{|y|<d_{U}(x_0)} \frac{ |y|^2 }{ |y|^{n+2s} } \,dy\\&=- \frac{n\delta }{2(1+{{\,\textrm{diam}\,}}(G_*)^2)} <0, \end{aligned}$$

a contradiction. $$\square $$

#### Corollary 1.37

(Mid-range comparison principle) Let $G\subseteq \Omega$ be open. Suppose $u\in USC(\overline{G_*})$, $v\in LSC(\overline{G_*})$ are respectively super- and sub-solutions to (8.1), i.e.

$$\begin{aligned}{\left\{ \begin{array}{ll} \mathcal {L}_{G_*}u \le f \le \mathcal {L}_{G_*}v &{} \text { in }G,\\ u\le v &{} \text { on }\overline{G_*}\setminus G, \end{array}\right. }\end{aligned}$$

in the viscosity sense, then

$$\begin{aligned} u\le v \quad \text { in }G. \end{aligned}$$

### Supremum of sub-solutions

Define the family of admissible sub-solutions as

$$\begin{aligned} \mathcal {A}:=\left\{ v\in USC(\overline{\Omega }): \mathcal {L}_{\Omega }v\le f \,\text { in}\, \Omega , v\le g \hbox { on}\ \partial \Omega \right\} . \end{aligned}$$

The pointwise supremum of all sub-solutions in $\mathcal {A}$ is defined as

8.2 $$\begin{aligned} u(x):=\sup _{v\in \mathcal {A}} v(x). \end{aligned}$$

We will prove that *u* is a viscosity solution by showing that $u^*=g$ on $\partial \Omega$ so that $u^*=u$, and then verify that $u_*$ is a super-solution so that, by comparison, $u_*=u$.

#### Proposition 1.38

(Perron’s method) The function *u* defined in (8.2) lies in $C(\overline{\Omega })$ and is a viscosity solution to (8.1).

#### Lemma 1.39

The USC envelope of *u* defined by (8.2) is a sub-solution in the interior, i.e.

$$\begin{aligned} \mathcal {L}_{\Omega }u^* \le f \qquad \text { in } \Omega . \end{aligned}$$

As a result, $\sup _{\overline{\Omega }}u^*\le C$, for $C>0$ depending only on *n*, *s*, $\left\| {f}\right\| _{L^\infty (\Omega )}$ and $\Omega$.

#### Proof

The proof is the same as [4, Lemma 4.15], except that the test function $\phi \in C^2$ is chosen such that $u-\phi$ attains its *global* maximum in $\overline{\Omega }$. $\square$

#### Lemma 1.40

The USC envelope of *u* defined by (8.2) satisfies the boundary condition, i.e.

$$\begin{aligned} u^*|_{\partial \Omega }=g\in C(\partial \Omega ). \end{aligned}$$

#### Proof

The proof is similar to [4, Proof of Theorem 4.17, Step 1], but a mid-range comparison is to be employed. Indeed, for each $x_0\in \partial \Omega$, let $r_0$ be as in Proposition 3.1 and define the barrier

$$\begin{aligned} w_{\varepsilon }^{\pm } :=g(x_0)\pm (\varepsilon +k_\varepsilon \varphi ^{(x_0)}) \qquad \text { in } \overline{B_{2r_0}(x_0)}. \end{aligned}$$

where $k_\varepsilon$, depending not only on $\varepsilon$ but also on *g*, $\Omega$ and $\sup _{\overline{\Omega }}{u^*}$, is chosen such that

$$\begin{aligned} w_{\varepsilon }^- \le u^* \le w_{\varepsilon }^+ \qquad \text { in } \overline{B_{2r_0}(x_0)} \setminus B_{r_0}(x_0) \supset \overline{B_{r_0}(x_0)_*}. \end{aligned}$$

By Proposition 3.1,

$$\begin{aligned} \mathcal {L}_{\Omega }w_{\varepsilon }^- = -k_\varepsilon \le \mathcal {L}_{\Omega } u^* \le k_\varepsilon =\mathcal {L}_{\Omega }w_{\varepsilon }^+ \qquad \text { in } B_{r_0}(x_0). \end{aligned}$$

By Corollary 8.6,

$$\begin{aligned} w_{\varepsilon }^- \le u^* \le w_{\varepsilon }^+ \qquad \text { in } B_{r_0}(x_0). \end{aligned}$$

In particular, since $\varphi ^{(x_0)} \in C(\overline{\Omega })$, there exists $\delta (\varepsilon )>0$ such that

$$\begin{aligned} g(x_0)-2\varepsilon \le u^* \le g(x_0)+2\varepsilon \qquad \text { in } B_{\delta (\varepsilon )}(x_0). \end{aligned}$$

This implies $\lim _{x_k\rightarrow x_0} u^*(x_k)=g(x_0)$ and hence $u^*|_{\partial \Omega }=g \in C(\partial \Omega )$. $\square$

#### Lemma 1.41

It holds that $u=u^* \in USC(\overline{\Omega })$.

#### Proof

By definition, $u\le u^*$. By Lemmas 8.8 and 8.9, $u^*\in \mathcal {A}$, so $u^* \le u$. $\square$

#### Lemma 1.42

Let *G* be an open subset of $\Omega$. Suppose $u\in USC(\overline{\Omega })$ satisfies

$$\begin{aligned} \mathcal {L}_{\Omega }u \le f \qquad \text { in } \Omega , \end{aligned}$$

in the viscosity sense, and $v\in C^2(G) \cap L^\infty (\overline{\Omega })$ satisfies pointwise

$$\begin{aligned} {\left\{ \begin{array}{ll} \mathcal {L}_{\Omega }v \le f &{} \text { in } G,\\ v \le u &{} \text { in } \overline{\Omega }\setminus G. \end{array}\right. } \end{aligned}$$

Then, the maximum $w=u\vee v$ is also a sub-solution in $\Omega$.

#### Proof

Suppose $x\in N_x\subset \Omega$ and $\phi \in C^2(N_x)\cap L^1(\Omega )$ is such that $w(x)=\phi (x)$ and $w\le \phi$ in $\overline{\Omega }$. We want to show that $\mathcal {L}_{\Omega }\phi (x) \le f(x)$. If $w(x)=u(x)$, then since $u\le w\le \phi$, the result follows from the fact that *u* is a viscosity sub-solution. If $w(x)=v(x) \ne u(x)$, then $x\in G$ and (using $v\le w\le \phi$) the pointwise computation also gives $\mathcal {L}_{\Omega }\phi (x) \le \mathcal {L}_{\Omega }v \le f$, as desired. $\square$

#### Lemma 1.43

The LSC envelope of *u* defined by (8.2) is a super-solution in the interior, i.e.

$$\begin{aligned} \mathcal {L}_{\Omega }u_* \ge f \qquad \text { in } \Omega . \end{aligned}$$

#### Proof

If $u_*$ is not a super-solution in $\Omega$, then there exists $x\in N_x\subset \Omega$, $\varphi \in C^2(N_x)\cap L^1(\overline{\Omega })$, such that $u(x)=\varphi (x)$, $u_*\ge \varphi$ in $\overline{\Omega }$, while $\mathcal {L}_{\Omega }\varphi (x)<f(x)$. By replacing $\varphi$ by ${{\tilde{\varphi }}}=\varphi -\varepsilon |\cdot -x|^2$ if necessary (where $\varepsilon$ depends on $\varphi$ and *f*), we can assume that $u_*>\varphi$ in $\overline{\Omega }\setminus \left\{ x\right\}$. By the continuity of $\mathcal {L}_{\Omega }\varphi$ and *f* at *x*, there exist $\delta ,\rho >0$ such that

$$\begin{aligned} \varphi +\delta<u_*\le u \quad \text { in } \overline{\Omega }\setminus B_{\rho }(x), \quad \text { and } \quad \mathcal {L}_{\Omega }\varphi < f \text { in } B_{\rho }(x). \end{aligned}$$

Now, define $u_\delta =u\vee (\varphi +\delta )$, which is a sub-solution in $\Omega$ due to Lemma 8.11. Now $u_\delta \in \mathcal {A}$ and so $u_\delta \le u$. But this means that $\varphi +\delta \le u$ in all of $\overline{\Omega }$ including $x_0$, a contradiction.

Suppose, on the contrary, that $u_*$ is not a super-solution in $\Omega$. Then there exists $x_0\in \Omega$ and $\varphi \in C(\overline{\Omega }) \cap C^2(B_{d_{\Omega }(x_0)}(x_0))$ such that $u_*(x_0)=\varphi (x_0)$, $u_* \ge \varphi$ in $\overline{\Omega }$, while $\mathcal {L}_{\Omega }\varphi (x_0)<f(x_0)$. By replacing $\varphi$ by ${\tilde{\varphi }}=\varphi -\varepsilon |x-x_0|^2$ if necessary, where $\varepsilon$ depends on $\varphi$ and *f*, we can assume that $u_*>\varphi$ in $\overline{\Omega } \setminus \left\{ x_0\right\}$. By continuity of $\varphi$, *f* and $\mathcal {L}_{\Omega }\varphi$ at $x_0$, there exists $\delta ,\rho >0$ such that

$$\begin{aligned} \varphi +\delta< u_* \le u \text { in } \overline{\Omega }\setminus B_{\rho }(x_0) \supset \overline{B_\rho (x_0)_*}\setminus B_{\rho }(x_0), \quad \text { and } \quad \mathcal {L}_{\Omega }\varphi < f \text { in } B_{\rho }(x_0). \end{aligned}$$

Now, define $u_\delta =u \vee (\varphi +\delta )$, which is a sub-solution in $\Omega$ due to Lemma 8.11. Now $u_\delta \in \mathcal {A}$ and so $u_\delta \le u$. But this means that $\varphi +\delta \le u$ in all of $\overline{\Omega }$ including $x_0$, a contradiction. $\square$

#### Lemma 1.44

It holds that $u=u_*\in LSC(\overline{\Omega })$.

#### Proof

By definition $u_*\le u$. In view of Lemmas 8.9 and 8.12, comparing $u_*$ to *u* via Corollary 8.6 gives $u\le u_*$. $\square$

#### Proof of Proposition 8.7

By Lemmas 8.9, 8.10 and 8.13, $u\in C(\overline{\Omega })$ is both a sub- and super-solution, and $u=g$ on $\partial \Omega$. $\square$

### Regularity

Since it suffices to obtain qualitative interior regularity, we compare to the (restricted) fractional Laplacian in $\mathbb {R}^n$ as in Lemma 2.4, and invoke the ccorresponding regualrity results in [3, Chapter 3].

#### Lemma 1.45

Let $u \in C(\overline{\Omega })$ be as in Proposition 8.7, with $f\in C^\alpha (\Omega )$. Then $u\in C^{2s+\alpha }(\Omega )$.

#### Proof

We verify that *u*, when extended continuously to a bounded function with compact support outside $\Omega$, is a viscosity solution to

8.3 $$\begin{aligned} (-\Delta )^su=F[u](x) \quad \text { in }\Omega , \end{aligned}$$

where *u* is and

8.4 $$\begin{aligned} F[u](x) = \dfrac{ c_{n,s} }{ C_{n,s} } \left( C_{n,s}\int _{B_{d(x)}^c} \dfrac{ u(x)-u(x+y) }{ |y|^{n+2s} } \,dy +f(x)d(x)^{2-2s} \right) . \end{aligned}$$

Recalling the definition of viscosity solution in [3, Chapter 3], suppose $x\in N_x\subset \Omega$ and $\phi \in L^1_{2\,s}(\mathbb {R}^n) \cap C^2(N_x)$ is such that

$$\begin{aligned} u(x)=\phi (x) \quad \text { and } \quad u\le \phi \text { in } \mathbb {R}^n. \end{aligned}$$

In particular, $\phi \in L^1(\Omega )$ and $u\le \phi$ in $\overline{\Omega }$. By Definition 8.3, $\mathcal {L}_{\Omega }\phi (x)\le f(x)$. This pointwise inequality rearranges to $(-\Delta )^s\phi \le F[\phi ](x)$. Hence, *u* is a viscosity sub-solution to (8.3)–(8.4). Similarly, *u* is also a viscosity super-solution. By bootstrapping the regularity result in [3, Chapter 3] (recall that *F*[*u*] is as regular as *u*, as in the proof of Lemma 2.4), $u\in C^{2s+\alpha }(\Omega )$. $\square$

#### Proof of Proposition 8.1

By Proposition 8.7, there exists a viscosity solution $u\in C(\overline{\Omega })$. By Lemma 8.14, $u\in C^{2\,s+\alpha }(\Omega )$, so it is also a classical solution. $\square$

#### Proof of Theorem 1.2

It follows immediately from Proposition 8.1 and Theorem 1.1. $\square$

## Data Availability

Data sharing not applicable to this article as no datasets were generated or analysed during the current study.

## Appendix A: Basic properties of $\mathcal {L}_\Omega$

We show that $\mathcal {L}_\Omega$ reduces to the classical Laplace operator at the boundary, with the choice of the normalization $C_{n,s}d(x)^{2-2s}$.

### Lemma A.1

(Limit operator) If $u\in C^{2,\beta }(\Omega )$ for some $\beta >0$, then

$$\begin{aligned} \mathcal {L}_\Omega u(x) \rightarrow -\Delta u(x), \quad \text { as }x\rightarrow \partial \Omega . \end{aligned}$$

### Proof

We compute

$$\begin{aligned}\begin{aligned} \mathcal {L}_{\Omega }u(x)&=-C_{n,s}d(x)^{2s-2} \text {P.V.}\,\int _{B_{d(x)}} \dfrac{ \frac{1}{2}D^2u(x)[y,y] +O\left( [D^2 u]_{C^\beta (\Omega )} |y|^{2+\beta } \right) }{ |y|^{n+2s} } \,dy\\&=-C_{n,s}d(x)^{2s-2} |\mathbb {S}^{n-1}| \int _0^{d(x)} \dfrac{ \Delta u(x)\frac{r^2}{2n} }{ r^{n+2s} } r^{n-1}\,dr +O\left( [D^2 u]_{C^\beta (\Omega )} d(x)^\beta \right) )\\&\rightarrow -C_{n,s}\dfrac{|\mathbb {S}^{n-1}|}{2n(2-2s)} \Delta u(x) =-\Delta u(x), \end{aligned}\end{aligned}$$

as $$d(x)\rightarrow 0$$. $$\square $$

A nice bound is available for $\mathcal {L}_\Omega$ on $C^{1,1}$ functions. We will need it only for $\delta$, a smooth function that agrees with $d_\Omega$ near the boundary.

### Lemma A.2

We have

$$\begin{aligned} \left| \mathcal {L}_\Omega \delta (x)\right| \le n\left[ { \nabla \delta }\right] _{C^{0,1}(B_{d(x)}(x))}, \quad \forall x\in \Omega . \end{aligned}$$

In particular, $\mathcal {L}_\Omega \delta$ is universally bounded.

### Proof

Since $\delta \in C^{1,1}(\overline{\Omega })$, by a Taylor expansion with quadratic error and (1.1),

$$\begin{aligned}\begin{aligned} |\mathcal {L}_\Omega \delta (x)| \le C_{n,s} d(x)^{2s-2} \int _{B_{d(x)}} \dfrac{ \frac{1}{2} \left[ { \nabla \delta }\right] _{C^{0,1}(B_{d(x)}(x))} |y|^2 }{ |y|^{n+2s} } \,dy =n\left[ {\nabla \delta }\right] _{C^{0,1}(B_{d(x)}(x))}. \end{aligned}\end{aligned}$$

$$\square $$

We collect the effect of translation and scaling on $L_\Omega$, since the operator depends heavily on the domain. When various domains are in consideration, we put the domain as a subscript.

Let $z\in \mathbb {R}^n$. For $u:\Omega \rightarrow \mathbb {R}$, define $u(\cdot ;z):\Omega -z\rightarrow \mathbb {R}$ by

$$\begin{aligned} u(x;z)=u(x+z). \end{aligned}$$

### Lemma A.3

(Translation) Let $u\in C^{2s+}(\Omega )$. For any $z\in \mathbb {R}^n$,

$$\begin{aligned} \mathcal {L}_{\Omega -z}u(x;z) =\mathcal {L}_{\Omega }u(x+z). \end{aligned}$$

### Proof

Since

$$\begin{aligned} d_{\Omega -z}(x)=d_{\Omega }(x+z) \quad \text { for }x\in \Omega -z, \end{aligned}$$

we have

$$\begin{aligned}\begin{aligned} \mathcal {L}_{\Omega -z}u(x;z)&=C_{n,s}d_{\Omega -z}(x)^{2s-2} \text {P.V.}\,\int _{B_{d_{\Omega -z}(x)}} \dfrac{ u(x;z)-u(x+y;z) }{ |y|^{n+2s} } \,dy\\&=C_{n,s}d_{\Omega }(x+z)^{2s-2} \text {P.V.}\,\int _{B_{d_{\Omega }(x+z)}} \dfrac{ u(x+z)-u(x+z+y) }{ |y|^{n+2s} } \,dy\\&=\mathcal {L}_{\Omega }(x+z). \end{aligned}\end{aligned}$$

$$\square $$

Let $r>0$. For $u:\Omega \rightarrow \mathbb {R}$, consider the rescaling $u_r:r^{-1}\Omega \rightarrow \mathbb {R}$ given by

$$\begin{aligned} u_r(x)=u(rx). \end{aligned}$$

### Lemma A.4

(Scaling) Let $u\in C^{2s+}(\Omega )$. For any $r>0$,

$$\begin{aligned} \mathcal {L}_{r^{-1}\Omega }u_r(x) =r^2 \mathcal {L}_{\Omega }u(rx). \end{aligned}$$

### Proof

Note that

$$\begin{aligned} d_{r^{-1}\Omega }(x)=r^{-1}d_{\Omega }(rx), \quad \text { for }x\in r^{-1}\Omega . \end{aligned}$$

Therefore

$$\begin{aligned}\begin{aligned} \mathcal {L}_{r^{-1}\Omega }u_r(x)&=C_{n,s}d_{r^{-1}\Omega }(x)^{2s-2} \text {P.V.}\,\int _{B_{d_{r^{-1}\Omega }(x)}} \dfrac{u_r(x)-u_r(x+y)}{|y|^{n+2s}} \,dy\\&=C_{n,s} r^{2-2s}d_{\Omega }(rx)^{2s-2} \text {P.V.}\,\int _{B_{r^{-1}d_{\Omega }(rx)}} \dfrac{ u(rx)-u(rx+ry) }{ r^{-n-2s}|ry|^{n+2s} } r^{-n}\,d(ry)\\&=r^2 \mathcal {L}_{\Omega }u(rx).\\ \end{aligned}\end{aligned}$$

$$\square $$

## Appendix B: An auxiliary function

Let

B.1 $$\begin{aligned} \begin{aligned} \psi (p,t)&= \dfrac{C_{n,s}}{2} t^{2s-2} \int _{|z|<t} \dfrac{ (1+z_n)^p+(1-z_n)^p-2 }{ |z|^{n+2s} } \,dz\\&= \dfrac{C_{n,s}}{2} \int _{|y|<1} \dfrac{ (1+ty_n)^p+(1-ty_n)^p-2 }{ t^2|y|^{n+2s} } \,dy. \end{aligned} \end{aligned}$$

Note that, by (1.1), $\psi (2,t)=2$ for all $t>0$.

### Lemma B.1

For $$p>0$$ and $$t\in [0,1]$$,

$$\begin{aligned} 0\le \psi _p(p,t) \le C. \end{aligned}$$

Consequently,

$$\begin{aligned} \left| \psi (p,t)-\psi (2,t) \right| \le C|p-2|. \end{aligned}$$

Here the constant *C* depends only on *n*, *s* and *p* and it remains bounded as $p\rightarrow 2$.

### Proof

It suffices to bound

$$\begin{aligned} \psi _p(p,t) = \dfrac{C_{n,s}}{2} \int _{|y|<1} \dfrac{ (1+ty_n)^p\log (1+ty_n) +(1-ty_n)^p\log (1-ty_n) }{ t^2|y|^{n+2s} } \,dy \ge 0. \end{aligned}$$

When $t\in (0,1/2)$ or $|y_n|<1/2$, we have $|ty_n|<1/2$ and so Taylor expansion gives

$$\begin{aligned}\begin{aligned}&\quad \; (1+ty_n)^p\log (1+ty_n) +(1-ty_n)^p\log (1-ty_n) \\&\le (1+Cty_n)(ty_n+Ct^2y_n^2) +(1+Cty_n)(-ty_n+Ct^2y_n^2)\\&\le Ct^2y_n^2. \end{aligned}\end{aligned}$$

When $t\in [1/2,1]$ and $|y_n|\ge 1/2$, we have also $|y|\ge 1/2$ so the integrand is bounded (by the boundedness of the function $x\mapsto x^p\log x$ on [0, 2]). so

$$\begin{aligned}\begin{aligned} \psi _p(p,t)&\le C+C\int _{|y_n|<1/2} \dfrac{ y_n^2 }{ |y|^{n+2s} } \,dy \le C.\\ \end{aligned}\end{aligned}$$

$$\square $$

### Lemma B.2

There exists $c>0$ depending only on *n* and *s* such that as $q\rightarrow 0^+$,

$$\begin{aligned} -\psi (q,t) \le cq+O(q^2), \end{aligned}$$

uniformly in $$t\in [0,1]$$.

### Proof

Using the Taylor expansion

$$\begin{aligned}&\quad \; (1+y)^q+(1-y)^q-2\\&=(\log (1+y)+\log (1-y))q +\frac{ (1+y)^{q_*}(\log (1+y))^2 +(1-y)^{q_*}(\log (1-y))^2 }{2}q^2\\&=\log (1-y^2)\cdot q+O(y^2q^2), \end{aligned}$$

we have

$$\begin{aligned} -\psi (q,t) = \left( \frac{C_{n,s}}{2}t^{2s-2} \int _{|y|<t} \frac{ \log (1-y^2) }{ |y|^{n+2s} } \,dy \right) q +O(q^2), \end{aligned}$$

where the error $O(q^2)$ is bounded independently of $t\in [0,1]$. To see that the coefficient of *q* remains strictly positive as $t\rightarrow 0$, we observe that for $|y|<1/2$, $\log (1-y^2) \le Cy^2$ and the homogeneity similar to (1.1). $\square$

## Appendix C: Boundary expansions

### Lemma C.1

Suppose $\Omega$ is $C^{1,1}$, $0\in \partial \Omega$ and $u\in C^{1,\gamma }(\Omega )$. Let $\nu (x)$ denotes the inward normal of the parallel surface containing *x*, and assume $\nu (0)=e_n$. Suppose

C.1 $$\begin{aligned} \dfrac{u(x)}{d(x)}=c_0+O(|x|^{\gamma }) \quad \text { as }x\rightarrow 0. \end{aligned}$$

Then

C.2 $$\begin{aligned} u(x)=c_0(x\cdot \nu (x))+O(|x|^{1+\gamma }) \quad \text { as }x\rightarrow 0. \end{aligned}$$

### Proof

Represent $\partial \Omega$ by a graph $x_n=\Phi (x')$ near $x=(x',x_n)=0$, then $\Phi (0)=|\nabla \Phi (0)|=0$ and

$$\begin{aligned} x\cdot \nu (x) =x_n+x\cdot (\nu (x)-e_n) =d(x)+O(\Phi (x))+O(|D^2\Phi (x)||x|^2) =d(x)+O(|x|^2). \end{aligned}$$

Suppose (C.1) holds. Then

$$\begin{aligned} u(x)=c_0d(x)+O(|x|^{\gamma }d(x)). \end{aligned}$$

Clearly $d(x)=O(|x|)$ as $x\rightarrow 0$. Thus (C.2) holds. $\square$
